# Hospital factors and metastatic surgery in colorectal cancer patients, a population-based cohort study

**DOI:** 10.1186/s12885-022-10005-8

**Published:** 2022-08-19

**Authors:** Malin Ljunggren, Caroline E. Weibull, Emma Rosander, Gabriella Palmer, Bengt Glimelius, Anna Martling, Caroline Nordenvall

**Affiliations:** 1 Department of Molecular Medicine and Surgery, Karolinska Institutet, Colorectal Surgery Unit, Karolinska University Hospital, Anna Steckséns gata 30A D2:05, 171 76 Solna Stockholm, Sweden; 2 Medical Unit of Trauma, Emergency Surgery and Orthopaedics, Karolinska University Hospital, Stockholm, Sweden; 3 Clinical Epidemiology Division, Department of Medicine, Karolinska Institutet, Stockholm, Sweden; 4 Department of Surgery and Urology, Danderyd Hospital, Stockholm, Sweden; 5 Department of Pelvic Cancer, GI Oncology and Colorectal Surgery Unit, Karolinska University Hospital, Stockholm, Sweden; 6 Department of Immunology, Genetics and Pathology, Uppsala University, Uppsala, Sweden

**Keywords:** Colorectal neoplasms, High-volume hospitals, Low-volume hospitals, Neoplasm metastasis, Secondary, Surgery, Survival, University hospitals

## Abstract

**Background:**

Only a limited proportion of patients with metastatic colorectal cancer (mCRC) receives metastatic surgery (including local ablative therapy). The aim was to investigate whether hospital volume and hospital level were associated with the chance of metastatic surgery.

**Methods:**

This national cohort retrieved from the CRCBaSe linkage included all Swedish adult patients diagnosed with synchronous mCRC in 2009–2016. The association between annual hospital volume of incident mCRC patients and the chance of metastatic surgery, and survival, were assessed using logistic regression and Cox regression models, respectively. Hospital level (university/non-university) was evaluated as a secondary exposure in a similar manner. Both uni- and multivariable (adjusted for sex, age, Charlson comorbidity index, year of diagnosis, cancer characteristics and socioeconomic factors) models were fitted.

**Results:**

A total of 1,674 (17%) out of 9,968 mCRC patients had metastatic surgery. High hospital volume was not associated with increased odds of metastatic surgery after including hospital level in the model, whereas hospital level was (odds ratio (OR) (95% confidence interval (CI)): 1.94 (1.68–2.24)). All-cause mortality was lower in university versus non-university hospitals (hazard ratio (95% CI): 0.83 (0.78–0.88)).

**Conclusions:**

Patients with mCRC initially cared for by a university hospital experienced a greater chance to receive metastatic surgery and had superior overall survival. High hospital volume in itself was not associated with a greater chance to receive metastatic surgery nor a greater survival probability. Additional efforts should be imposed to provide more equal care for mCRC patients across Swedish hospitals.

**Supplementary Information:**

The online version contains supplementary material available at 10.1186/s12885-022-10005-8.

## Background

Colorectal cancer (CRC) is the second most common cause of cancer death worldwide [1]. Overall survival (OS) for patients with metastatic CRC (mCRC) has improved substantially thanks to developments in medical oncology and because a larger number of initially unresectable patients with metastatic disease can be offered surgical conversion [2, 3]. Moreover, local ablative treatment in combination with systemic treatment compared to systemic treatment only, is associated with superior OS for patients with unresectable liver metastases [4]. Metastatic surgery (including non-surgical local ablative treatments) in combination with primary tumour resection can offer selected patients the chance of cure.

In a universal healthcare system, such as that in Sweden, one of the most important principles is equal access to care [5]. The healthcare system in Sweden is decentralised into six healthcare regions and 21 regions, which are responsible for managing and prioritising their own healthcare resources. All six healthcare regions have one university hospital, except one that has two. A few university hospitals have multiple physical locations. Surgery of metastases for mCRC is limited to a few hospitals, mainly the university hospitals. Cytoreductive surgery with hyperthermic intraperitoneal chemotherapy (HIPEC) is further centralised to four university hospitals. Previous studies on mCRC have found that hospital factors, such as hospital level (university hospital/non-university hospital) and geographic location, are associated with the chance of receiving surgery for liver metastases [6–13], peritoneal metastases [14], pulmonary metastases [15] and mCRC overall [16, 17].

An increased chance of metastatic surgery for mCRC has also been associated with being discussed at a multidisciplinary team (MDT) conference [18, 19]. This has contributed to the recommendation that as of 2016, *all* CRC patients in Sweden should be discussed at MDT conferences, and in the presence of liver metastases, with a liver surgeon present [20]. In the recommendations from 2008 CRC patients with limited metastases were to be considered potentially curable and therefore further assessed in a multidisciplinary setting [21].

Increased hospital and surgeon volume are associated with improved outcomes for CRC patients undergoing surgery [22–25]. In the presence of metastases an equivalently important quality of care measure is related to the patients who do not undergo surgery. To explore whether differences in provided care exist, all patients with mCRC need to be identified and included. Studies of pancreatic cancer patients have shown that hospital volume based on all incident cases, both surgically and non-surgically treated, is associated to a higher frequency of surgery and/ or chemoradiation [26].

Sweden has several high-quality registers including the Swedish colorectal cancer register (SCRCR), which had a coverage of 98.5% of colon adenocarcinomas and 98.8% of rectal adenocarcinomas in 2008–2016 [27]. All Swedish citizens and residents are registered in the Swedish Population Register and have unique personal identity numbers, which make linkage between registries possible. This provides an excellent opportunity to study all patients diagnosed with mCRC, regardless of what treatment they have later received. To our knowledge, the potential association between hospital volume of incident mCRC patients and the chance of having metastatic surgery has not been reported previously.

In this large nationwide Swedish study, variations in the proportion of mCRC patients receiving metastatic surgery between hospitals of different volume and level was evaluated. Being discussed at an MDT conference was explored as a mediator. It was hypothesised that hospital volume and level of hospital were associated with the chance of metastatic surgery and with OS.

## Methods

### Register data

This study is based on the Colorectal Cancer Database (CRCBaSe), a mega-linkage originating from the SCRCR, which was further linked to national registers at the National Board of Welfare and Statistics Sweden. The SCRCR was used to identify patients, to get information on the registering hospital and on surgery of metastases and primary tumour, and to retrieve data on potential confounders and descriptive variables. When a patient is diagnosed with CRC, the hospital is responsible for registering the patient in the SCRCR and the managing hospital is automatically recorded when starting a registration in the SCRCR.

The Swedish Cancer Register (SCR), which dates back to 1958, was used to retrieve data on other cancer diagnoses and include data on previous CRC diagnoses not captured by the SCRCR, to ensure that only the first CRC diagnosis was included. Information from the Cause of Death Register was used to retrieve information on cause and date of death. The In- and Out-Patient Register was used to obtain information on comorbidities and to define presence of metastatic disease, receival of primary tumour resection and surgery of lung, liver and peritoneal metastases (Supplementary Table 1). From the Register of the Total Population, data on civil status and migration was extracted. From the Longitudinal Integrated Database for Health Insurance and Labour Market Studies data on disposable income per family unit and educational level were extracted. Civil status and highest achieved level of education were defined at the time of CRC diagnosis, while for income, information on the year prior to CRC diagnosis was used. If data was unavailable for the mentioned year, data from 0–3 years before year of diagnosis was used instead.

### Inclusion and exclusion criteria

**Fig. 1:**
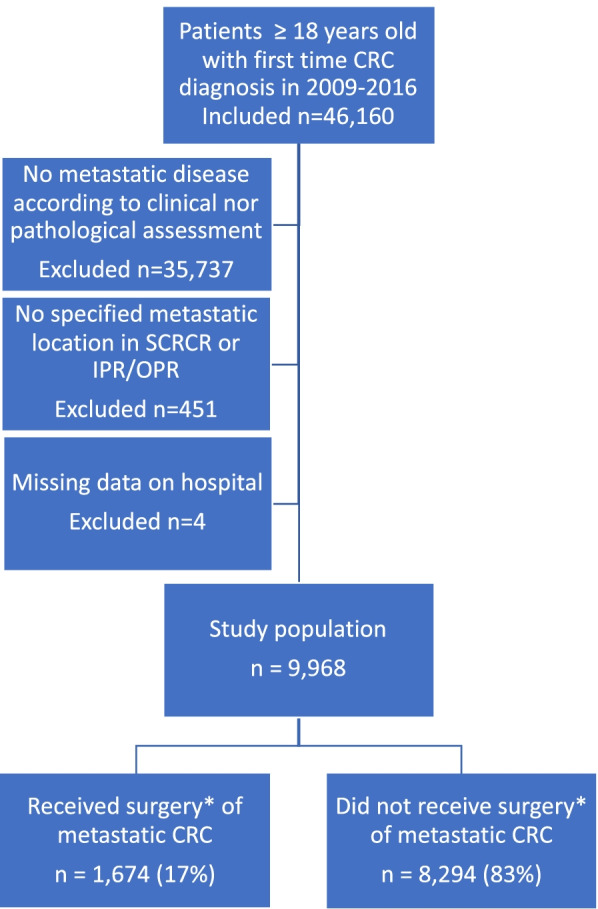
Flowchart showing study criteria for patients diagnosed with first-time synchronous metastatic colorectal cancer in 2009–2016. *Within predefined time window: 90 days before diagnosis to 270 days after diagnosis. Abbreviations: CRC=colorectal cancer, IPR=In-Patient Register,OPR=Out-Patient Register, SCR=Swedish Cancer Register, SCRCR=Swedish Colorectal Cancer Register

All Swedish adult patients (≥ 18 years old) with a first-time CRC diagnosis in the SCRCR during 2009–2016, with no previous diagnosis of CRC in SCR, were included (*n* = 46,160). The following exclusions criteria were applied: no presence of synchronous metastatic disease according to a clinical or pathological assessment (*n* = 35,737), no specified metastatic location (*n* = 451), and missing data on registering hospital (*n *= 4). The final study population consisted of 9,968 patients (Fig. 1).

**Fig 2 A-D:**
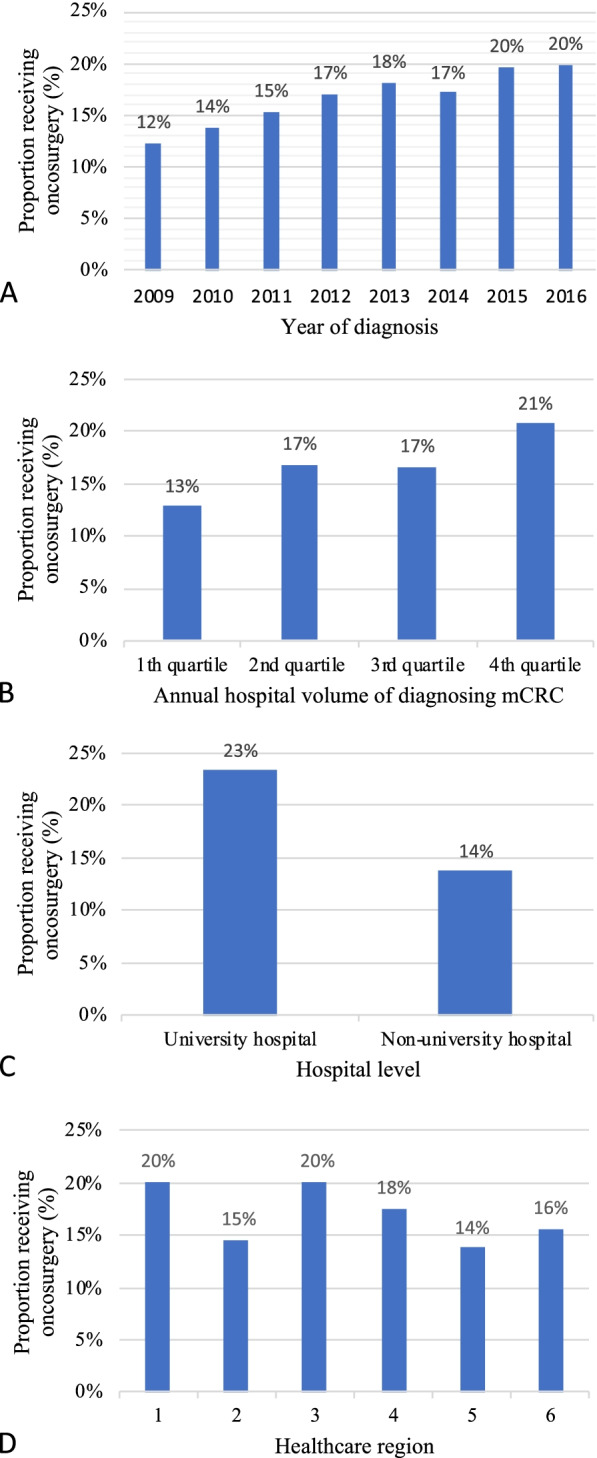
Metastatic surgery by **A**) year **B**) hospital volume, **C**) hospital level and **D**) healthcare region. *Including 9,968 patients with synchronous metastatic colorectal cancer (mCRC). The figures show the proportion receiving metastatic surgery between 90 days before mCRC diagnosis and 270 days after. P-value from trend test was* < *0.001 for A-B and P-value from chi square test was* < *0.001 for C-D.*

### Exposures and outcomes

Our primary exposure was annual hospital volume of incident mCRC patients, i.e., the total number of patients diagnosed with mCRC at a specific hospital during a given year. The annual hospital volume was divided into quartiles (≤ 25^th^, > 25^th^ to ≤ 50^th^, > 50^th^ to ≤ 75^th^, and > 75^th^). As such, the same hospital could be exposed as a 1^st^ quartile hospital one year and then fall into another quartile of hospital volume another year. Additionally, level of hospital (university or non-university) and its association with odds of metastatic surgery was investigated. Our primary outcome was receiving metastatic surgery (yes/no), which was defined as receiving surgical resection including a non-surgical locally ablative procedure of liver, and/or peritoneal, and/or lung metastases within 90 days prior to and 270 days after the date of primary CRC diagnosis date (Supplementary Table 2). This time window was chosen to encompass the majority of treatments for synchronous metastases, but not include treatment for metachronous metastases. Furthermore, it was required that the patient underwent primary tumour resection, either before, simultaneously or after the surgery of the metastases, to be defined as having received the primary outcome. Our secondary outcome was OS from date of diagnosis.

### Statistical methods

Frequencies and proportions of patient and tumour characteristics were calculated separately within hospital volume quartiles. The proportion of patients receiving metastatic surgery by year of diagnosis, hospital volume quartiles, hospital level, and healthcare region, were visualised using bar charts. Chi-squared tests were used for formal testing of differences in distributions, and logistic regression was applied to evaluate trends.

Crude and adjusted logistic regression models were fitted to estimate odds ratios (ORs) with 95% confidence intervals (CIs) of receiving metastatic surgery. The first multivariable model included one exposure (hospital volume or level) and adjusted for sex (female, male), age (18–64 years, 65–79 years and ≥ 80 years old), Charlson comorbidity index (CCI) [28] (0, 1 and ≥ 2), year of diagnosis (continuous 2009–2016), location of the primary tumour (right colon, transverse colon, left colon, and unknown location in colon or rectum), clinical tumour (cT) stage (1–3, 4, X), clinical nodal (cN) stage (0, 1–2, X), extent of metastatic disease (single location/ multiple locations), civil status (married/registered partner, or not married), highest commenced educational level (primary school, secondary school, or higher education) and disposable income per family unit (divided into four quartiles per year of diagnosis). A second multivariable model was constructed including both exposures (hospital volume and level) and adjusted for the variables mentioned above. An additional multivariable model also included the potential mediator MDT (yes/no). Tests for interactions between hospital volume and hospital level, and hospital level and MDT conference, were performed using a Wald test. Missing data were included in the adjusted models by use of the missing-indicator method [29].

For the survival analyses, the patients were followed from date of diagnosis until date of death, migration, or 31^st^ December 2017, whichever occurred first. The underlying time scale was time since diagnosis. The Kaplan–Meier method was used to calculate OS proportions, and the log rank test to test for survival differences, by hospital volume and hospital level. Cox regression models were used to estimate hazard ratios (HRs) with 95% CIs of the all-cause mortality rate for the four hospital volume quartiles and hospital level. Both univariable and multivariable models were fitted, where the latter adjusted for the same variables as described in the multivariable logistic regression models above. The proportional hazard assumption was formally tested using the Schoenfeld residuals.

A sensitivity analysis was performed using a delayed entry model, in which follow-up started at 270 days after diagnosis. Patients who died (*n* = 3,964) or were administratively censored (*n* = 11) during these 270 days were not included in this analysis (n,_included_ = 5,993). The Kaplan–Meier method was used to calculate OS proportions, and the log rank test to test for survival differences, by whether the patients received metastatic surgery or not.

The significance level was set to 5%. Statistical analyses were done using Stata (StataCorp. 2017. Stata Statistical Software: Release 16. College Station, TX: StataCorp, LLC).

### Ethical approval

Ethical approval was given by the Regional Board of the Ethical Committee in Stockholm, Sweden (DNR: 2014/71–31, 2018/328–32, 2021–00,342) and the study was conducted in accordance with the ethical standards described.

## Results

A total of 9,968 adult patients diagnosed with mCRC in Sweden 2009–2016 met the study criteria (Fig. 1). Of these, 1,674 (17%) had surgery of their metastases during the pre-defined time window around diagnosis and primary tumour resection (procedures performed are shown in Supplementary Table 2). The proportion of patients with mCRC who had metastatic surgery increased from 12% in 2009 to 20% in 2016 (Fig. 2A, p for trend < 0.001).

The annual volumes of incident mCRC patients at hospitals in the 1^st^, 2^nd^, 3^rd^, and 4^th^ quartiles were 1–20, 21–31, 32–46, and 47–103 patients per year, respectively. The proportion of patients receiving surgery for mCRC increased from 13% in the 1^st^ quartile to 21% in the 4^th^ quartile (Fig. 2B, p for trend < 0.001). The proportion receiving metastatic surgery was 23% of patients initially cared for by a university hospital compared to 14% at non-university hospitals (Fig. 2C,* p* < 0.001). During the period, 20% of patients diagnosed with mCRC received surgery in healthcare region 1 and 3 compared with 14% and 15% in healthcare region 5 and 2, respectively (Fig. 2D, *p* < 0.001).

### Patient, hospital and tumour characteristics

Patients managed at high-volume hospitals (4^th^ quartile) were younger at diagnosis, had reached a higher level of education and a higher disposable income (Table 1). There were no differences in CCI (Table 1). Approximately half of the patients underwent resection of the primary tumour, a third had liver metastases only, and about half of the patients had metastatic disease at multiple locations (Table 2). Around 24% (*n* = 327/1,359) of the patients who underwent primary tumour resection and liver surgery had synchronous primary tumour and liver surgery. The proportion of patients initially cared for at a university hospital ranged between 3% in the 1^st^ quartile and 77% in the 4^th^ quartile of hospital volume. Data on race/ ethnicity of study participants was not available.

**Table 1:** Patient characteristics according to annual hospital volume of patients with metastatic colorectal cancer

		First quartile *n* = 2,328	Second quartile *n *= 2,591	Third quartile *n *= 2,531	Fourth quartile *n* = 2,518	All *n* = 9,968	*P*-value*
Sex	Male	1,325 (57%)	1,455 (56%)	1,386 (55%)	1,400 (56%)	5,566 (56%)	0.466
	Female	1,003 (43%)	1,136 (44%)	1,145 (45%)	1,118 (44%)	4,402 (44%)	
Age	Median (years)	71	70	71	69	70	
	18–64 years	631 (27%)	784 (30%)	757 (30%)	889 (35%)	3,061 (31%)	< 0.001
	65–79 years	1,182 (51%)	1,265 (49%)	1,221 (48%)	1,169 (46%)	4,837 (49%)	
	≥ 80 years	515 (22%)	542 (21%)	553 (22%)	460 (18%)	2,070 (21%)	
Charlson comorbidity index	0	1,469 (63%)	1,600 (62%)	1,523 (60%)	1,543 (61%)	6,135 (62%)	0.378
	1	229 (10%)	287 (11%)	290 (11%)	264 (10%)	1,070 (11%)	
	≥ 2	630 (27%)	704 (27%)	718 (28%)	711 (28%)	2,763 (28%)	
Year of diagnosis	2009–2012	1,228 (55%)	1,359 (52%)	1,198 (47%)	980 (39%)	4,825 (48%)	< 0.001
	2013–2016	1,040 (45%)	1,232 (48%)	1,333 (53%)	1,538 (61%)	5,143 (52%)	
Civil status **	Married/registered partner	1,258 (54%)	1,416 (55%)	1,329 (53%)	1,309 (52%)	4,654 (47%)	0.348
	Not married: never married, divorced, widowed	1,070 (46%)	1,175 (45%)	1,201 (47%)	1,208 (48%)	5,312 (53%)	
	Missing	0 (0%)	0 (0%)	1 (0%)	1 (0%)	2 (0%)	
Highest level of commenced education **	Primary school (maximum 9 years)	909 (39%)	1,011 (39%)	965 (38%)	775 (31%)	3,660 (37%)	< 0.001
	Secondary school (3–4 additional years)	945 (41%)	1,011 (39%)	993 (39%)	1,039 (41%)	3,988 (40%)	
	Higher education	459 (20%)	531 (20%)	550 (22%)	680 (27%)	2,220 (22%)	
	Missing	14 (1%)	38 (1%)	24 (1%)	24 (1%)	100 (1%)	
Disposable income per family unit divided into annual quartiles. ***	1st quartile	676 (29%)	653 (25%)	624 (25%)	543 (22%)	2,496 (25%)	< 0.001
	2nd quartile	609 (26%)	646 (25%)	648 (26%)	584 (23%)	2,487 (25%)	
	3rd quartile	569 (24%)	685 (26%)	633 (25%)	605 (24%)	2,492 (25%)	
	4th quartile	474 (20%)	607 (23%)	625 (25%)	785 (31%)	2,491 (25%)	
	Missing	0 (0%)	0 (0%)	1 (0%)	1 (0%)	2 (0%)	

Includes 9,968 patients diagnosed with metastatic colorectal cancer in 2009–2016 in Sweden

^*^ From chi square test

^**^ Year of/up to 3 years before if missing

^***^Year before diagnosis or up to 3 years before or year of if missing

**Table 2:** Hospital and tumour characteristics according to annual hospital volume of patients with metastatic colorectal cancer

		First quartile *n* = 2,328	Second quartile *n *= 2,591	Third quartile *n *= 2,531	Fourth quartile *n* = 2,518	All *n* = 9,968	*P*-value*
Hospital level	University	79 (3%)	492 (19%)	669 (26%)	1,928 (77%)	3,168 (32%)	< 0.001
	Non-university	2,249 (97%)	2,099 (81%)	1,862 (74%)	590 (23%)	6,800 (68%)	
Hospital region	1	221 (9%)	370 (14%)	635 (25%)	741 (29%)	1,967 (20%)	< 0.001
	2	505 (22%)	608 (23%)	869 (34%)	273 (11%)	2,255 (23%)	
	3	370 (16%)	449 (17%)	231 (9%)	0 (0%)	1,050 (11%)	
	4	390 (17%)	459 (18%)	407 (16%)	763 (30%)	2,019 (20%)	
	5	390 (17%)	401 (15%)	288 (11%)	741 (29%)	1,820 (18%)	
	6	452 (19%)	304 (12%)	101 (4%)	0 (0%)	857 (9%)	
Primary tumour location	Right colon	615 (26%)	637 (25%)	643 (25%)	676 (27%)	2,568 (26%)	< 0.001
	Transverse colon	314 (13%)	292 (11%)	316 (12%)	306 (12%)	1,227 (12%)	
	Left colon	743 (32%)	761 (29%)	732 (29%)	673 (27%)	2,912 (29%)	
	Unknown location in colon	21 (1%)	19 (1%)	9 (0%)	19 (1%)	68 (1%)	
	Rectum	625 (27%)	882 (34%)	831 (33%)	844 (34%)	3,193 (32%)	
Pretherapeutic multidisciplinary team conference	Yes	1,507 (65%)	1,967 (76%)	1,950 (77%)	2,011 (80%)	7,435 (75%)	< 0.001
	No	812 (35%)	621 (24%)	570 (23%)	498 (20%)	2,510 (25%)	
	Missing	8 (0%)	3 (0%)	3 (0%)	9 (0%)	23 (0%)	
Resection of primary tumour	No	1,128 (48%)	1,349 (52%)	1,397 (55%)	1,322 (53%)	5,196 (52%)	< 0.001
	Yes	1,199 (52%)	1,242 (48%)	1,135 (45%)	1,196 (47%)	4,772 (48%)	
cT	1–3	659 (28%)	959 (37%)	824 (33%)	824 (34%)	3,264 (33%)	< 0.001
	4	564 (24%)	757 (29%)	735 (29%)	795 (32%)	2,849 (29%)	
	X	619 (27%)	485 (19%)	500 (20%)	611 (24%)	2,218 (22%)	
	Missing	486 (21%)	290 (15%)	472 (19%)	288 (11%)	1,637 (16%)	
cN	0	450 (19%)	531 (20%)	413 (16%)	401 (16%)	1,795 (18%)	< 0.001
	1–2	1,099 (47%)	1,434 (55%)	1,521 (60%)	1,362 (54%)	5,414 (54%)	
	X	660 (28%)	543 (21%)	540 (21%)	702 (28%)	2,447 (25%)	
	Missing	119 (5%)	83 (3%)	57 (2%)	53 (2%)	312 (3%)	
Metastatic location	Liver	744 (32%)	913 (35%)	908 (36%)	805 (32%)	3,370 (34%)	< 0.001
	Lung	205 (9%)	176 (7%)	170 (7%)	128 (5%)	679 (7%)	
	Peritoneum	62 (3%)	70 (3%)	85 (3%)	111 (4%)	328 (3%)	
	Other	73 (3%)	93 (4%)	83 (3%)	105 (4%)	354 (4%)	
	Multiple locations	1,244 (53%)	1,339 (52%)	1,285 (51%)	1,369 (54%)	5,237 (53%)	

Includes 9,968 patients diagnosed with metastatic colorectal cancer in 2009–2016 in Sweden

^*^ From chi square test

*Abbreviations*: *cN* (clinical nodal status), *CT* (clinical tumour status)

### Metastatic surgery

**Fig. 3:**
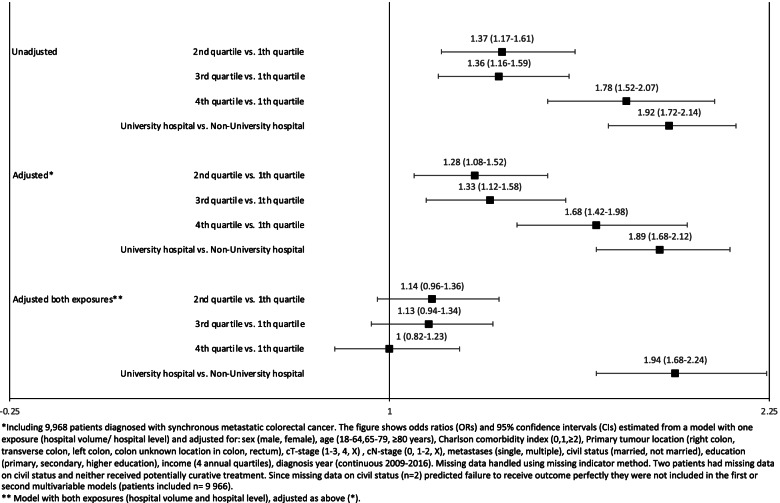
Forest plot of annual hospital volume, hospital level and the chance of receiving metastatic surgery

The chance of receiving metastatic surgery increased with hospital volume, compared to the 1^st^ quartile; 2^nd^ quartile OR (95% CI): 1.28 (1.08–1.52), 3^rd^ quartile OR (95% CI): 1.33 (1.12–1.58), 4^th^ quartile OR (95% CI): 1.68 (1.42–1.98), from adjusted models (Fig. 3 and Supplementary Table 3). The chance of metastatic surgery was higher in university hospitals than non-university hospitals (OR (95% CI): 1.89 (1.68–2.12). After including both exposures, hospital volume and hospital level, there was no association between hospital volume and outcome. The same analysis revealed that patients managed at a university hospital had increased odds of having metastatic surgery (OR (95% CI): 1.94 (1.68–2.24)) also after adjustment for hospital volume (Fig. 3 and Supplementary Table 3). There was a significant interaction between hospital volume and hospital level (p from Wald test < 0.001). This can be interpreted as the effect of hospital volume was significantly different for university hospitals compared with non-university hospitals.

### MDT and temporal trends

The proportion discussed at a pretherapeutic MDT conference increased from 60% in 2009 to 86% in 2016 (*p*-value from trend test < 0.001). In 2009, 73% of mCRC patients at university hospitals were discussed at MDT conferences and 54% at non-university hospitals (*p* < 0.001). The proportion being discussed at MDT conferences were similar in 2016, 86% vs 85% at university and non-university hospitals, respectively (*p* = 0.777). In light of the diminishing differences in the proportion being discussed at an MDT by hospital level at the end of the study period, we investigated if the same was evident for differences in metastatic surgery. The proportion receiving metastatic surgery at a university hospital compared to a non-university hospital was 18% vs. 10% in 2009 (*p* < 0.001) and 28% vs 16% in 2016 (*p* < 0.001). When investigating how the OR for the association between hospital level and receiving metastatic surgery was altered by including the potential mediator MDT, the results were mainly unchanged and university hospital remained significantly associated with the chance of metastatic surgery (OR (95% CI): 1.90 (1.64–2.19)), whereas hospital volume remained not associated (Supplementary Table 3). This can be interpreted as the full effect of hospital level is not mediated by MDT, but is a direct effect or mediated by some other factor. Furthermore, MDT was an independent predictor of receiving metastatic surgery in the model (OR (95% CI): 1.54 (1.30–1.82)). The effect of being discussed at an MDT was different between different hospital levels (p from Wald test < 0.001), but MDT was positively associated with receiving metastatic surgery at both university hospitals and non-university hospitals.

### Overall survival

**Figs 4:**
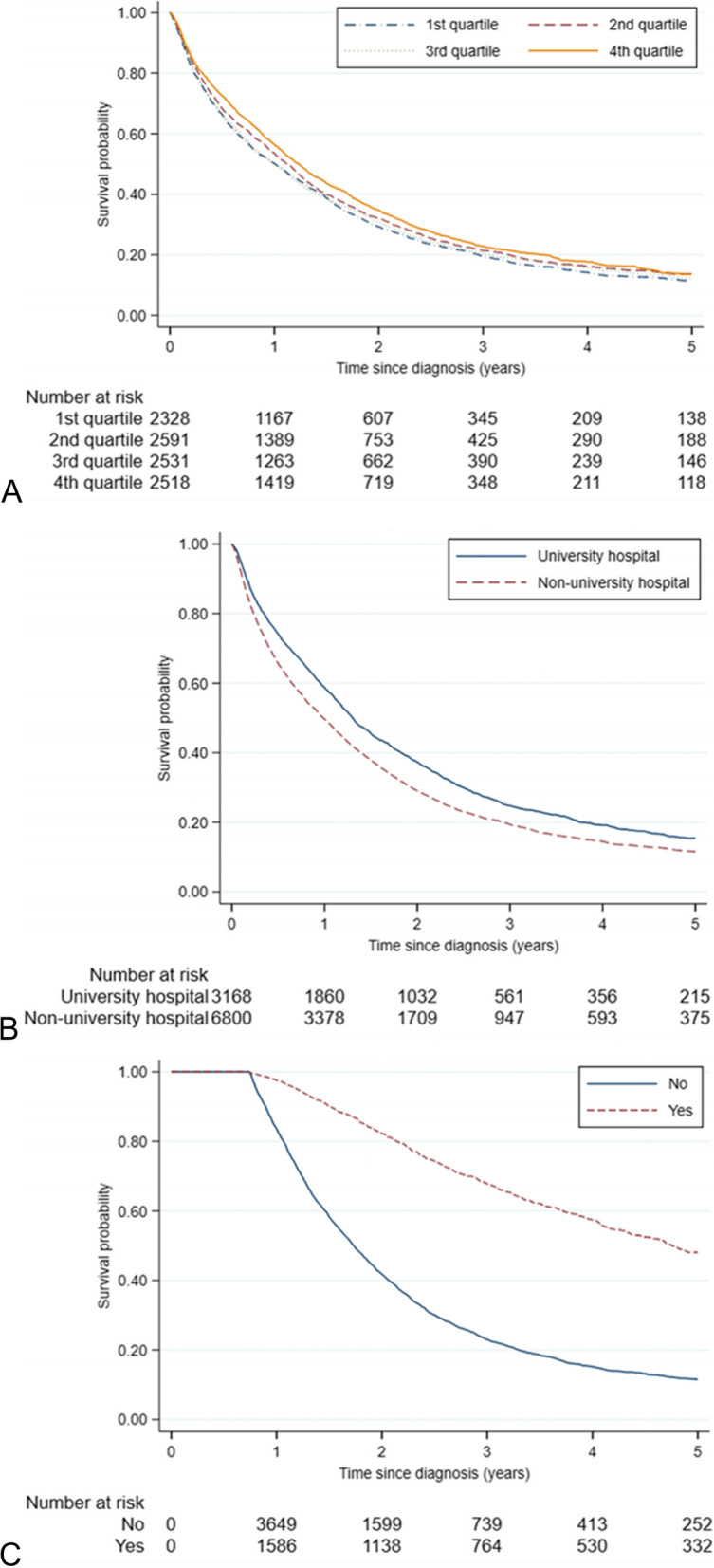
**A-C** Cumulative overall survival (OS) estimates by **A**) hospital volume, **B**) hospital level, **C**) metastatic surgery. *Kaplan–Meier estimates of OS including 9,968 patients followed from diagnosis of synchronous metastatic colorectal cancer. The stratification by metastatic surgery was based on a delayed entry of patients in model to 270 days post diagnosis (n* = *5,993). P-value from log-rank test was p *= *0.001 for A and p* < *0.001 for B and C.*

The 1- and 5-years OS were slightly higher for patients managed at hospitals in the 4^th^ volume quartile (56% and 14%, respectively) compared with patients managed at hospitals in the 1^st^ volume quartile (50% and 11%, respectively, Fig. 4A-C). High hospital volume was associated with a decreased all-cause mortality rate (4^th^ versus 1^st^ quartile of hospital volume, adjusted HR (95% CI): 0.92 (0.86–0.98), Table 3 and Supplementary Table 3). When further adjustment was made for hospital level the association between hospital volume and all-cause mortality disappeared (Table 3 and Supplementary Table 3).

**Table 3:** Cox regression model estimates of all-cause mortality hazard ratios (HRs) with 95% confidence intervals (CIs)

		**Univariable**	**First model **^**1**^	**Second model**^**2**^
		HR^a^ (95% CI)	HR^a^ (95% CI)	HR^a^ (95% CI)
Hospital mCRC case load	1^st^ quartile	1	1	1
	2^nd^ quartile	**0.93 (0.87–0.99)**	1.01 (0.95–1.07)	1.04 (0.98–1.11)
	3^rd^ quartile	0.97 (0.92–1.04)	1.02 (0.96–1.09)	**1.07 (1.00–1.14)**
	4^th^ quartile	**0.87 (0.82–0.93)**	**0.92 (0.86–0.98)**	1.06 (0.98–1.14)
Hospital level	Non-university	1	1	1
	University	**0.81 (.78–0.85)**	**0.85 (0.81–0.89)**	**0.83 (0.78–0.88)**

Model includes 9,968 patients with synchronous colorectal cancer metastases

^a^Adjusted for time since diagnosis

^1^One exposure (hospital volume/ hospital level) and adjusted for sex (male, female), age (categorized: 18–64,65–79, ≥ 80 years), Charlson comorbidity index (0,1, ≥ 2), Primary tumour location (right colon, transverse colon, left colon, colon unknown location in colon, rectum), cT (1–3, 4, X) cN, (0, 1–2, X), metastases (single, multiple), civil status (married, not married), education (primary, secondary, higher education), annual income (quartiles), year of diagnosis. Missing data handled using missing indicator method

^2^Model of both exposures, adjusted as above

Similarly, there were differences in OS based on whether the managing hospital was a university hospital or not. The 1- and 5-year OS were 59% vs 50% and 15% vs 12% for patients managed at a university hospital vs. a non-university hospital, respectively (Fig. 4A-C). Survival was higher in patients managed at a university hospital in comparison with a non-university hospital (HR (95% CI): 0.83 (0.78–0.88), results from second model including both exposures). Neither the effect of hospital volume nor hospital level violated the proportionality assumption (*p*-value from test of proportional hazards > 0.05).

In the sensitivity analysis with delayed entry, the 1- and 5-year OS estimates were 97% and 48%, respectively for patients who received metastatic surgery, and 84% and 12% respectively in the group who did not receive metastatic surgery (Fig. 4C).

## Discussion

In this large nationwide cohort study of almost 10,000 patients with synchronous mCRC 17% of patients had surgery of their primary tumour and metastases. The most important hospital characteristic was management at a university hospital, where patients experienced almost a doubled chance of receiving metastatic surgery and an improved survival. The proportion receiving metastatic surgery varied significantly by healthcare region.

To the best of our knowledge, annual hospital volume of incident patients with mCRC has not previously been associated with whether or not metastatic surgery is performed. However, patients managed at a university hospital are known to experience an increased likelihood of having surgery for colorectal liver metastases [7, 8, 13] and peritoneal metastases [14]. Our results clearly show that the associations between hospital volume and metastatic surgery were essentially explained by hospital level. In Sweden, centralisation of most metastatic surgery analysed in this study has been to the university hospitals. In Sweden, specific cancer centres/hospitals are not present, whereas this is rather common in other healthcare systems, where they may or may not be connected to university hospitals. Further, metastatic surgeries may also be performed in non-university hospitals. Therefore, the results regarding hospital level probably holds the highest external validity in comparison with countries with a similar healthcare organisation, chiefly the other Nordic countries but also countries like the Netherlands. Hospital factors e.g. presence of a liver-centre on site [6, 30], or a HIPEC-centre on site [14], hospitals volume of liver resections [9], and management at a high-volume hospital of treatments for mCRC [16], have been associated with an increased likelihood of receiving metastatic site surgery. The finding that patients diagnosed at university hospitals in Sweden are more likely to receive metastatic surgery may be mediated through many factors which are more common in the university hospitals. Even if our finding of hospital level associated with metastatic surgery may not be immediately be applicable to other healthcare environments, we can more confidently conclude that hospital volume on its own is not associated with more metastatic surgery and better outcomes for mCRC patients.

The reasons why patients at non-university hospitals are less likely to be referred to metastatic surgery are probably multifactorial, reflecting differences in opinion or even knowledge of the treating physicians or the patient’s wish not to be referred. One explanation for the association seen is that interventional clinical studies are common at university hospitals, a factor that has been associated with improved outcome for CRC patients [31]. Patients with mCRC have improved OS when treated at academic programmes/ hospitals compared with community programmes/district hospitals [7, 8, 14, 32]. Another important factor is whether the metastases are deemed resectable, upfront or after conversion therapy, or not. Although all regions in Sweden but a few have a dedicated clinical oncology department with specialists in both radiation and medical oncology, the university hospitals have larger oncology departments with more specialists also devoted to research. It is possible that patients at university hospitals receive more active conversion chemotherapy resulting in greater possibilities for, e.g., liver surgery. Previous studies have also illustrated that the assessment of resectability of liver metastasis is highly dependent on the observer, where referring physicians can incorrectly consider some metastatic lesions as non-resectable [33] and liver specialists are more prone to deem metastases as resectable [11, 34]. During the 8-year period analysed here, a marked change in what is considered resectable has occurred. This is probably a major reason for the increasing temporal trend in the proportion of metastatic surgery noted, but could also be responsible for the differences seen between healthcare regions. Such differences tend to decrease with time, although we could not detect any indications of this. Individual surgeons, medical oncologists and interventional radiologists at the different university hospitals may also have affected the results. In summary, the importance of avoiding individual decisions and instead making use of MDT conferences, with specialised oncologists and subspecialised surgeons present, must be stressed.

MDTs are positively associated with the chance of metastatic surgery for mCRC [18, 19]. To explore whether presence of MDTs could be a mediator for the observed differences in metastatic surgery by hospital level we performed an explanatory model adjusting for MDT. Our results showed that the elevated chance of metastatic surgery for patients managed at a university hospital remained, indicating that there are other reasons for the dissimilarities observed. It is possible that the quality of the discussion varied at different hospital levels, but this information is not possible to obtain. An alternative to local or even regional MDTs could be centralised repeated assessments of resectability, which increased the proportion eligible for surgery of CRC liver metastases in a Finnish material [34]. Future studies are needed to evaluate if this is sufficient to diminish the demonstrated variability in provided care by hospital level, or between different healthcare regions, for mCRC patients.

### Strengths and weaknesses

The greatest strength of this national cohort study is its size and quality of data. Thanks to the recently established CRCBaSe, we had access to high-quality data from SCRCR and several other large national registries. The previous Swedish study on selection for metastatic surgery for mCRC in 2007–2011 was based on the SCRCR and restricted to data on liver resections only [8]. In the current study we could complement the SCRCR data with data from several other registers e.g., treatment codes from the patient register and therefore also study metastatic surgery of the lungs and peritoneum. Another strength is that we could ascertain that the patients did not have a previous CRC diagnosis as far back as 1958. Moreover, by linkage to national patient registries, we could limit our study population to patients with at least one defined metastatic location, yielding high internal validity. In terms of external validity, the nationwide approach increased generalisability. We believe the results to be applicable to countries with similar hospital systems, especially those that have a similar centralisation of metastatic surgeries to university hospitals. In countries with specialised cancer centres outside of the university organisation, the conclusions regarding the benefits of being managed at university hospitals may be more difficult to transfer.

The primary weakness of this study is its confinement to available register data. Detailed data on factors related to treatment strategy, such as tumour burden at each metastatic location, would have been preferrable. Unfortunately, this has not been registered and is almost impossible to obtain retrospectively given the size of the study. Patients at high volume hospitals were younger and could therefore be deemed more suitable for the most demanding surgeries for mCRC. In addition, patients at high volume hospitals had higher socioeconomic status, a factor that has been associated with the chance of receiving surgery for mCRC [6, 7, 9, 10, 17, 30, 35]. However, the association between university hospital and the chance to receive metastatic surgery withheld after adjustment for mentioned confounders such as age, tumour characteristics, civil status, income and level of education. We acknowledge that there is a possibility of residual confounding. There may also be a risk of referral bias, if so, it is probably more pronounced in metropolitan areas where several hospitals are present within a short geographical distance. In the majority of cases, we believe the patients were referred to the closest hospital irrespective of tumour burden and comorbidities.

## Conclusions

This study indicates that patients with mCRC receive different surgical care in Sweden. Initial management at a university hospital was associated with a greater chance of receiving metastatic surgery for patients with synchronous mCRC, and improved survival. High hospital volume in itself was not associated with a greater chance to receive metastatic surgery nor a greater survival probability. These results show that additional efforts should be imposed in order to provide more equal care for mCRC patients.

## Supplementary Information

**Additional file 1: Supplementary table 1.** Treatment codes as described by the National Board of Health and Welfare in Sweden used to identify potentially curative treatments of metastases and primary tumour, translated from Swedish.*Defined as non-surgical treatment.** Supplementary table 2.** Additional treatment-associated patient characteristics for 9,968 patients with synchronous metastatic colorectal cancer (mCRC) according to the managing hospitals’ annual incident number of patients with mCRC divided into 4 quartiles. *Data unspecified on whether the neoadjuvant treatment was given for the primary tumour or the metastases or both. ** A total of 46 patients who underwent surgery for liver metastases (of which one had combination metastatic surgery) received solely non-surgical locally ablative therapy (percutaneous destruction or embolisation treatment) and four patients with lung metastases had solely non-surgical treatment (stereotactic body radiation, SBRT). The solely non-surgical locally ablative treatments constituted 3% (*n*=50/1797) of the patients treated with metastatic surgery. Abbreviations: *ASA* (American Society of Anaesthesiologists), *pN* (pathological nodal status), *pT* (pathological tumour status).** Supplementary table 3.** Summary table of results. All multivariable models were adjusted for sex (male, female), age (18-64,65-79, ≥80 years), Charlson comorbidity index (0,1,≥2), Primary tumour location (right colon, transverse colon, left colon, colon unknown location in colon, rectum), cT-stage (1-3, 4, X) , cN-stage (0, 1-2, X), metastases (single, multiple), civil status (married, not married), education (primary, secondary, higher education), income (4 annual quartiles), diagnosis year (continuous 2009-2016). Missing data handled using missing indicator method. Multivariable logistic regression models included 9,966 patients. Multivariable cox regression models included 9,968 patients. Abbreviations: CI: confidence interval, HR: hazard ratio, MDT: multidisciplinary team conference, OR: odds ratio, qt: quartile.

## Data Availability

The study is based on depersonalized data which cannot be shared by the authors due to the instructions of the ethical permission. For further questions regarding the data availability we recommend contacting the board of CRCBaSe (email: crcbase@mmk.ki.se).

## Abbreviations

CCI: Charlson comorbidity index
CI: Confidence interval
cN: Clinical nodal
CRC: Colorectal cancer
CRCBaSe: Colorectal cancer database
cT: Clinical tumour
HIPEC: Hyperthermic intraperitoneal chemotherapy
HR: Hazard ratio
mCRC: Metastatic colorectal cancer
MDT: Multidisciplinary team
OR: Odds ratio
OS: Overall survival
SCR: Swedish cancer register
SCRCR: Swedish colorectal cancer register

## References

[1] Sung H, Ferlay J, Siegel RL, Laversanne M, Soerjomataram I, Jemal A, et al. Global cancer statistics 2020: GLOBOCAN estimates of incidence and mortality worldwide for 36 cancers in 185 countries. CA Cancer J Clin. 2021;71(3):209–49. 33538338 10.3322/caac.21660

[2] Cremolini C, Loupakis F, Antoniotti C, Lupi C, Sensi E, Lonardi S, et al. FOLFOXIRI plus bevacizumab versus FOLFIRI plus bevacizumab as first-line treatment of patients with metastatic colorectal cancer: updated overall survival and molecular subgroup analyses of the open-label, phase 3 tribe study. Lancet Oncol. 2015;16(13):1306–15. 26338525 10.1016/S1470-2045(15)00122-9

[3] Tomasello G, Petrelli F, Ghidini M, Russo A, Passalacqua R, Barni S. FOLFOXIRI plus bevacizumab as conversion therapy for patients with initially unresectable metastatic colorectal cancer: A systematic review and pooled analysis. JAMA Oncol. 2017;3(7): e170278. 28542671 10.1001/jamaoncol.2017.0278 PMC5824228

[4] Ruers T, VanCoevorden F, Punt CJ, Pierie JE, Borel-Rinkes I, Ledermann JA, et al. Local treatment of unresectable colorectal liver metastases: Results of a randomized phase II trial. J Natl Cancer Inst. 2017;109(9):djx015. 28376151 10.1093/jnci/djx015 PMC5408999

[5] Swedish Code of Statues. SFS 2017:30 Hälso- och sjukvårdslag (Health and Medical Services Act). https://www.riksdagen.se/sv/dokument-lagar/dokument/svensk-forfattningssamling/halso--och-sjukvardslag_sfs-2017-30.

[6] Fenton HM, Taylor JC, Lodge JPA, Toogood GJ, Finan PJ, Young AL, et al. Variation in the use of resection for colorectal cancer liver metastases. Ann Surg. 2019;270(5):892–8. 31567507 10.1097/SLA.0000000000003534 PMC6867670

[7] Sell NM, Shafique N, Lee H, Lee GC, Tanabe KK, Ferrone CR, et al. Socioeconomic determinants of the surgical treatment of colorectal liver metastases. Am J Surg. 2020;220(4):952–7. 32107013 10.1016/j.amjsurg.2020.02.019

[8] Noren A, Eriksson HG, Olsson LI. Selection for surgery and survival of synchronous colorectal liver metastases; a nationwide study. Eur J Cancer. 2016;53:105–14. 26702764 10.1016/j.ejca.2015.10.055

[9] Raoof M, Jutric Z, Haye S, Ituarte PHG, Zhao B, Singh G, et al. Systematic failure to operate on colorectal cancer liver metastases in California. Cancer Med. 2020;9(17):6256–67. 32687265 10.1002/cam4.3316 PMC7476837

[10] Morris EJ, Forman D, Thomas JD, Quirke P, Taylor EF, Fairley L, et al. Surgical management and outcomes of colorectal cancer liver metastases. Br J Surg. 2010;97(7):1110–8. 20632280 10.1002/bjs.7032

[11] Young AL, Adair R, Culverwell A, Guthrie JA, Botterill ID, Toogood GJ, et al. Variation in referral practice for patients with colorectal cancer liver metastases. Br J Surg. 2013;100(12):1627–32. 24264786 10.1002/bjs.9285

[12] ’t Lam-Boer J, Al Ali C, Verhoeven RH, Roumen RM, Lemmens VE, Rijken AM, et al. Large variation in the utilization of liver resections in stage IV colorectal cancer patients with metastases confined to the liver. Eur J Surg Oncol. 2015;41(9):1217–25. 10.1016/j.ejso.2015.05.014 26095702

[13] ’t Lam-Boer J, van der Stok EP, Huiskens J, Verhoeven RH, Punt CJ, Elferink MA, et al. Regional and inter-hospital differences in the utilisation of liver surgery for patients with synchronous colorectal liver metastases in the Netherlands. Eur J Cancer. 2017;71(109):16. 10.1016/j.ejca.2016.10.026 27988444

[14] Rovers KP, Simkens GA, Vissers PA, Lemmens VE, Verwaal VJ, Bremers AJ, et al. Survival of patients with colorectal peritoneal metastases is affected by treatment disparities among hospitals of diagnosis: A nationwide population-based study. Eur J Cancer. 2017;75:132–40. 28222307 10.1016/j.ejca.2016.12.034

[15] Turner NH, Wong HL, Field K, Wong R, Shapiro J, Yip D, et al. Novel quality indicators for metastatic colorectal cancer management identify significant variations in these measures across treatment centers in Australia. Asia Pac J Clin Oncol. 2015;11(3):262–71. 25871458 10.1111/ajco.12355

[16] Krell RW, Regenbogen SE, Wong SL. Variation in hospital treatment patterns for metastatic colorectal cancer. Cancer. 2015;121(11):1755–61. 25640016 10.1002/cncr.29253 PMC4592280

[17] Healy MA, Pradarelli JC, Krell RW, Regenbogen SE, Suwanabol PA. Insurance status and hospital payer mix are linked with variation in metastatic site resection in patients with advanced colorectal cancers. Dis Colon Rectum. 2016;59(11):1047–54. 27749480 10.1097/DCR.0000000000000684 PMC5119894

[18] Segelman J, Singnomklao T, Hellborg H, Martling A. Differences in multidisciplinary team assessment and treatment between patients with stage IV colon and rectal cancer. Colorectal Dis. 2009;11(7):768–74. 18662241 10.1111/j.1463-1318.2008.01648.x

[19] Chen CH, Hsieh MC, Lao WT, Lin EK, Lu YJ, Wu SY. Multidisciplinary team intervention associated with improved survival for patients with colorectal adenocarcinoma with liver or lung metastasis. Am J Cancer Res. 2018;8(9):1887–98. 30323980 PMC6176172

[20] Nationellt vårdprogram. Tjock- och ändtarmscancer (Swedish National Cancer Strategy in Colorectal Cancer). Version 2,0. Stockholm: Regionala cancercentrum i samverkan (Regional Cancer Centres); 2016.

[21] Nationellt vårdprogram. Tjock- och ändtarmscancer (Swedish National Cancer Strategy in Colorectal Cancer). Version 1,0. Umeå: Regionala cancercentrum i samverkan (Regional Cancer Centres); 2008.

[22] Rogers SO, Wolf RE, Zaslavsky AM, Wright WE, Ayanian JZ. Relation of surgeon and hospital volume to processes and outcomes of colorectal cancer surgery. Ann Surg. 2006;244(6):1003–11. 17122626 10.1097/01.sla.0000231759.10432.a7 PMC1856632

[23] Liu CJ, Chou YJ, Teng CJ, Lin CC, Lee YT, Hu YW, et al. Association of surgeon volume and hospital volume with the outcome of patients receiving definitive surgery for colorectal cancer: A nationwide population-based study. Cancer. 2015;121(16):2782–90. 25892632 10.1002/cncr.29356

[24] van Gijn W, Gooiker GA, Wouters MW, Post PN, Tollenaar RA, van de Velde CJ. Volume and outcome in colorectal cancer surgery. Eur J Surg Oncol. 2010;36(Suppl 1):S55-63. 20615649 10.1016/j.ejso.2010.06.027

[25] Martling A, Cedermark B, Johansson H, Rutqvist LE, Holm T. The surgeon as a prognostic factor after the introduction of total mesorectal excision in the treatment of rectal cancer. Br J Surg. 2002;89(8):1008–13. 12153626 10.1046/j.1365-2168.2002.02151.x

[26] Bilimoria KY, Bentrem DJ, Ko CY, Tomlinson JS, Stewart AK, Winchester DP, et al. Multimodality therapy for pancreatic cancer in the U.S. : utilization, outcomes, and the effect of hospital volume. Cancer. 2007;110(6):1227–34. 10.1002/cncr.22916 17654662

[27] Moberger P, Sköldberg F, Birgisson H. Evaluation of the Swedish Colorectal Cancer Registry: an overview of completeness, timeliness, comparability and validity. Acta Oncol. 2018;57(12):1611–21. 30477372 10.1080/0284186X.2018.1529425

[28] Ludvigsson JF, Appelros P, Askling J, Byberg L, Carrero JJ, Ekström AM, et al. Adaptation of the Charlson comorbidity index for register-based research in Sweden. Clin Epidemiol. 2021;13:21–41. 33469380 10.2147/CLEP.S282475 PMC7812935

[29] Groenwold RH, White IR, Donders AR, Carpenter JR, Altman DG, Moons KG. Missing covariate data in clinical research: when and when not to use the missing-indicator method for analysis. CMAJ. 2012;184(11):1265–9. 22371511 10.1503/cmaj.110977 PMC3414599

[30] Vallance AE, van der Meulen J, Kuryba A, Braun M, Jayne DG, Hill J, et al. Socioeconomic differences in selection for liver resection in metastatic colorectal cancer and the impact on survival. Eur J Surg Oncol. 2018;44(10):1588–94. 29895508 10.1016/j.ejso.2018.05.024

[31] Downing A, Morris EJ, Corrigan N, Sebag-Montefiore D, Finan PJ, Thomas JD, et al. High hospital research participation and improved colorectal cancer survival outcomes: a population-based study. Gut. 2017;66(1):89–96. 27797935 10.1136/gutjnl-2015-311308 PMC5256392

[32] Atallah C, Oduyale O, Stem M, Eltahir A, Almaazmi HH, Efron JE, et al. Are academic hospitals better at treating metastatic colorectal cancer? Surg. 2021;169(2):248–56. 10.1016/j.surg.2020.05.023 32680747

[33] Krell RW, Reames BN, Hendren S, Frankel TL, Pawlik TM, Chung M, et al. Surgical referral for colorectal liver metastases: A population-based survey. Ann Surg Oncol. 2015;22(7):2179–94. 25582739 10.1245/s10434-014-4318-x PMC4631547

[34] Isoniemi H, Uutela A, Nordin A, Lantto E, Kellokumpu I, Ovissi A, et al. Centralized repeated resectability assessment of patients with colorectal liver metastases during first-line treatment: prospective study. Br J Surg. 2021;108(7):817–25. 33749772 10.1093/bjs/znaa145 PMC10364914

[35] Tabrizian P, Overbey J, Carrasco-Avino G, Bagiella E, Labow DM, Sarpel U. Escalation of socioeconomic disparities among patients with colorectal cancer receiving advanced surgical treatment. Ann Surg Oncol. 2015;22(5):1746–50. 25388060 10.1245/s10434-014-4220-6

