# Correction: Hospital factors and metastatic surgery in colorectal cancer patients, a population-based cohort study

**DOI:** 10.1186/s12885-025-14339-x

**Published:** 2025-05-23

**Authors:** Malin Ljunggren, Caroline E. Dietrich, Emma Rosander, Gabriella Palmer, Bengt Glimelius, Anna Martling, Caroline Nordenvall

**Affiliations:** 1 Department of Molecular Medicine and Surgery, Karolinska Institutet, Colorectal Surgery Unit, Karolinska University Hospital, Anna Steckséns Gata 30 A D2:05, Solna Stockholm, 171 76 Sweden; 2 Medical Unit of Trauma, Emergency Surgery and Orthopaedics, Karolinska University Hospital, Stockholm, Sweden; 3 Clinical Epidemiology Division, Department of Medicine, Karolinska Institutet, Stockholm, Sweden; 4 Department of Surgery and Urology, Danderyd Hospital, Stockholm, Sweden; 5 Department of Pelvic Cancer, GI Oncology and Colorectal Surgery Unit, Karolinska University Hospital, Stockholm, Sweden; 6 Department of Immunology, Genetics and Pathology, Uppsala University, Uppsala, Sweden

**Correction: BMC Cancer 22, 907 (2022)**

**https://doi.org/10.1186/s12885-022–10005-8**

Following publication of the original article [1], the authors identified an error in the original code to calculate the hospital volume quartiles. The error led to the effect that the quartiles were calculated based on the distribution of annual hospital volumes across the study period instead of calculations year by year. This led to ubiquitous but very slight changes throughout the manuscript. The effect size of the main results is essentially unchanged, and the statistical significance of the results is, thus, untouched. Two changes are noted. Firstly, the annual volumes of incident mCRC patients at hospitals in the 1st, 2nd, 3rd, and 4th quartiles now overlap with 1–26, 18–37, 28–53, 39–103 patients per year, respectively (previously 1–20, 21–31,32–46, 47–103). Secondly, in the patient characteristics table (Table 1) the annual hospital volume quartiles had significantly different proportions regarding the variable “year of diagnosis” (2009–2012/ 2013–2016). The p-value indicate that the proportion changed from statistically significantly different to non-significantly (p = 0.248) different. The affected Tables (Table 1, Table 2, Table 3), Figures (Fig. 3, Fig. 4) and Supplementary material (Supplementary Table 2 and Supplementary Table 3) were remade and all updated with the correct numbers per quartile, correct odds ratios or hazard ratios and confidence intervals. The authors regret their mistake.

Incorrect Fig. 3 is given below.

**Fig. 3:**
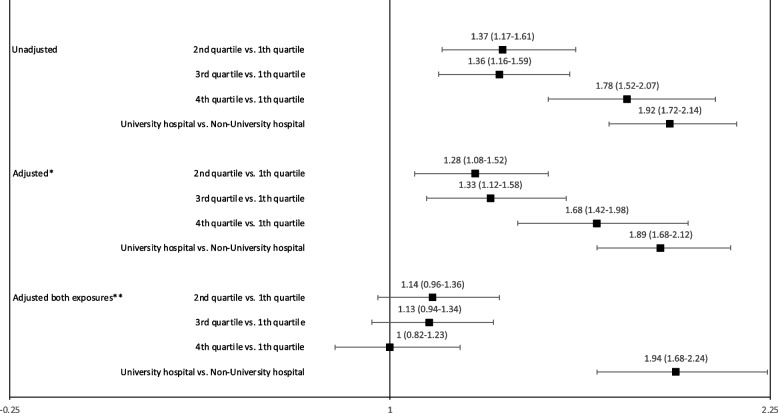
Forest plot of annual hospital volume, hospital level and the chance of receiving metastatic surgery

Correct Fig. 3 is given below.

**Fig. 3:**
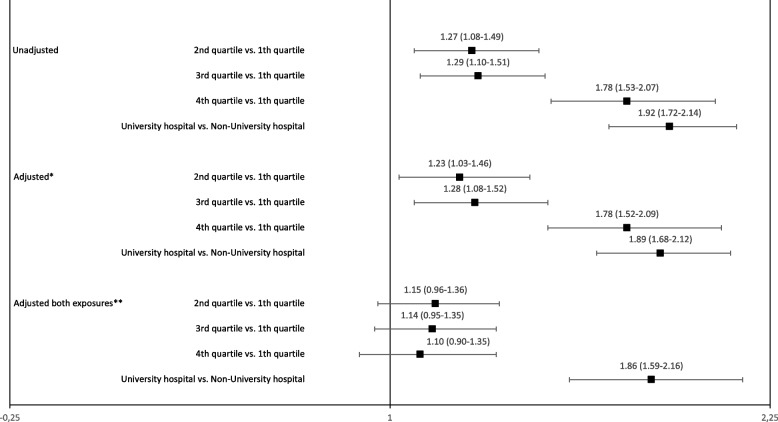
Forest plot of annual hospital volume, hospital level and the chance of receiving metastatic surgery

Incorrect Fig. 4 is given below.

**Fig. 4:**
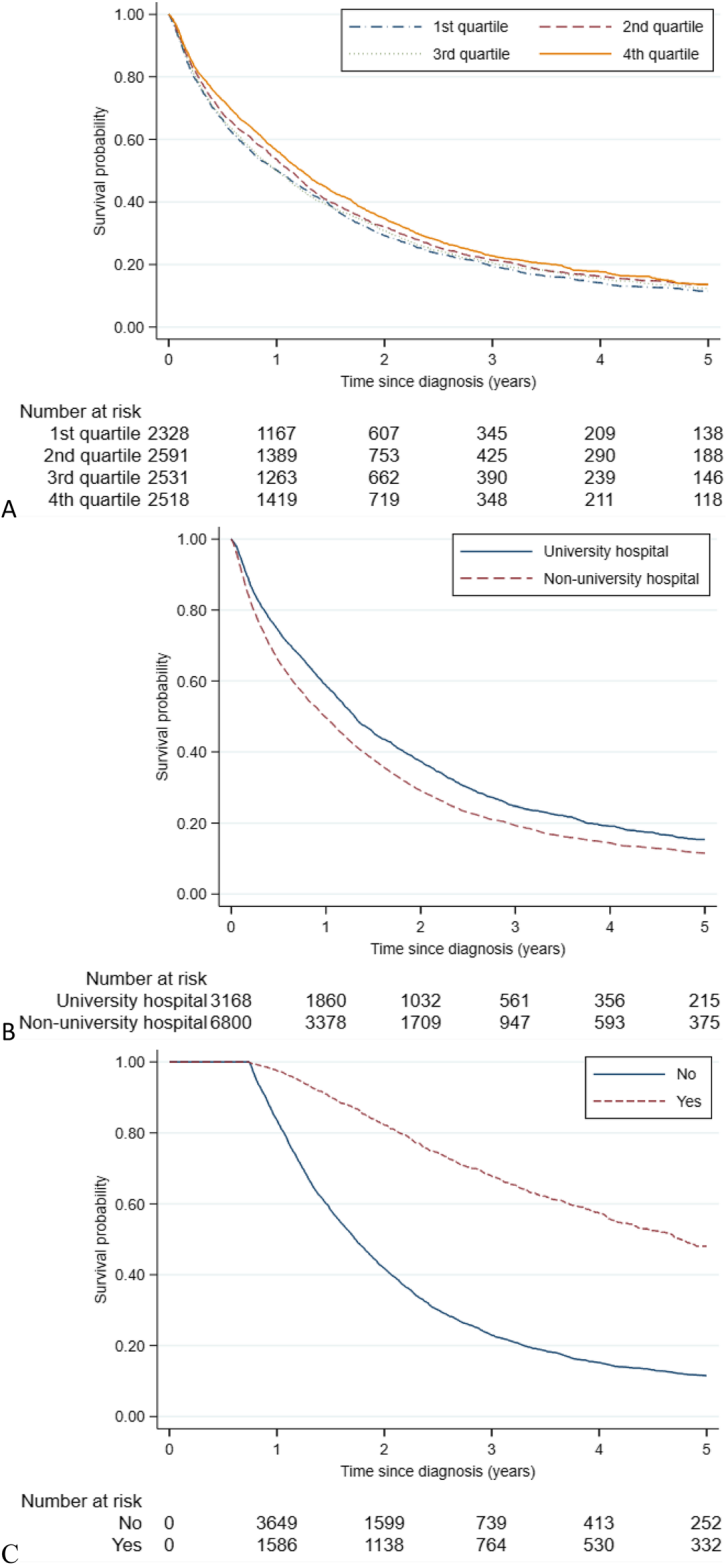
Cumulative overall survival (OS) estimates by **A**) hospital volume, **B**) hospital level, **C**) metastatic surgery. *Kaplan-Meier estimates of OS including 9,968 patients followed from diagnosis of synchronous metastatic colorectal cancer. The stratification by metastatic surgery was based on a delayed entry of patients in model to 270 days post diagnosis (n=5,993). P-value from log-rank test was p=0.001 for A and p<0.001 for B and C*

Correct Fig. 4 is given below.

**Fig. 4:**
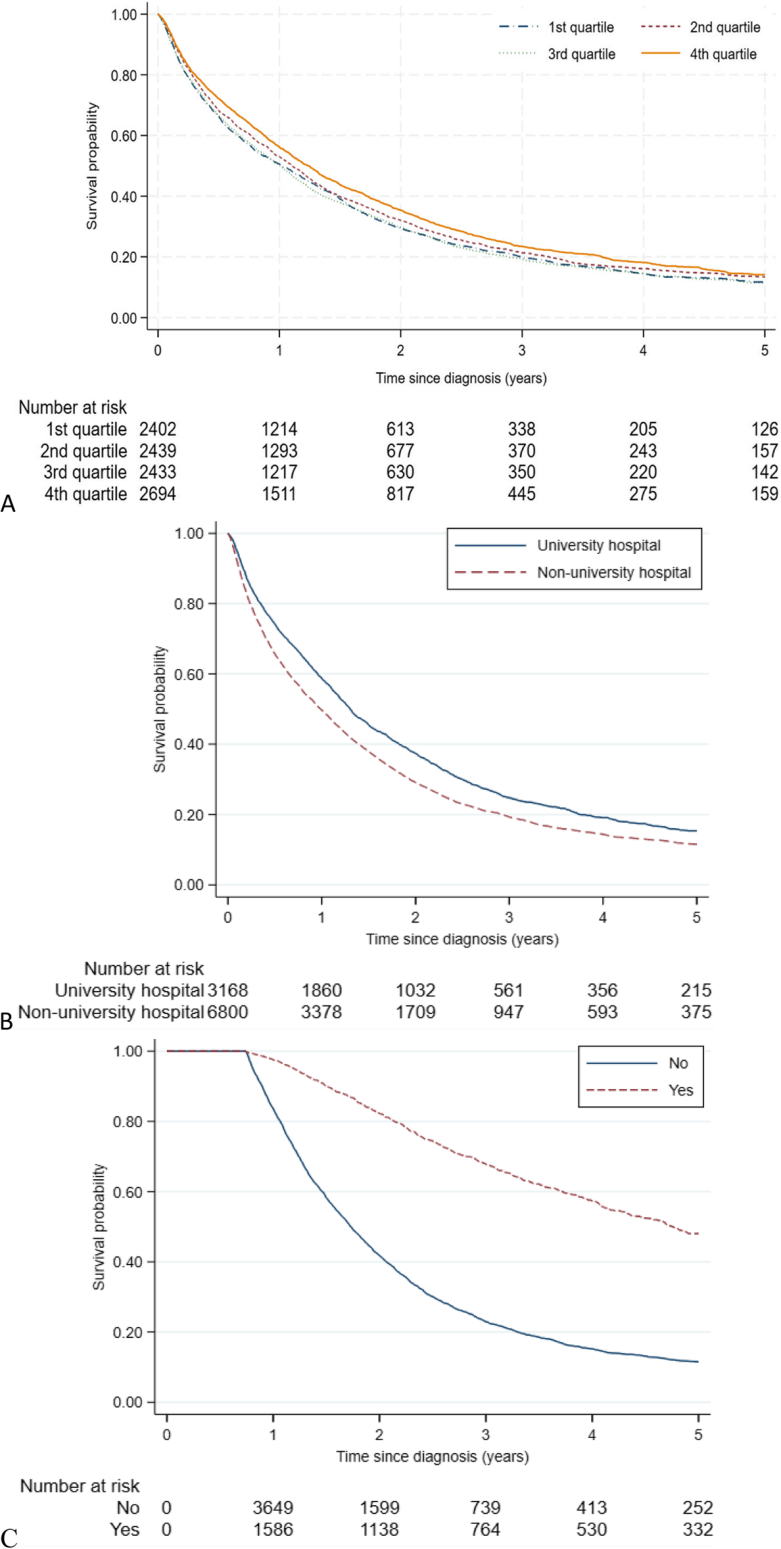
Cumulative overall survival (OS) estimates by **A**) hospital volume, **B**) hospital level, **C**) metastatic surgery. *Kaplan-Meier estimates of OS including 9,968 patients followed from diagnosis of synchronous metastatic colorectal cancer. The stratification by metastatic surgery was based on a delayed entry of patients in model to 270 days post diagnosis (n=5,993). P-values from log-rank test were p<0.001 for A, B and C*

The incorrect Table 1 is given below.

**Table 1:** Patient characteristics according to annual hospital volume of patients with metastatic colorectal cancer

		First quartile *n* = 2,328	Second quartile *n* = 2,591	Third quartile *n* = 2,531	Fourth quartile *n* = 2,518	All *n* = 9,968	*P*-value*
Sex	Male	1,325 (57%)	1,455 (56%)	1,386 (55%)	1,400 (56%)	5,566 (56%)	0.466
	Female	1,003 (43%)	1,136 (44%)	1,145 (45%)	1,118 (44%)	4,402 (44%)	
Age	Median (years)	71	70	71	69	70	
	18–64 years	631 (27%)	784 (30%)	757 (30%)	889 (35%)	3,061 (31%)	< 0.001
	65–79 years	1,182 (51%)	1,265 (49%)	1,221 (48%)	1,169 (46%)	4,837 (49%)	
	≥ 80 years	515 (22%)	542 (21%)	553 (22%)	460 (18%)	2,070 (21%)	
Charlson comorbidity index	0	1,469 (63%)	1,600 (62%)	1,523 (60%)	1,543 (61%)	6,135 (62%)	0.378
	1	229 (10%)	287 (11%)	290 (11%)	264 (10%)	1,070 (11%)	
	≥ 2	630 (27%)	704 (27%)	718 (28%)	711 (28%)	2,763 (28%)	
Year of diagnosis	2009–2012	1,228 (55%)	1,359 (52%)	1,198 (47%)	980 (39%)	4,825 (48%)	< 0.001
	2013–2016	1,040 (45%)	1,232 (48%)	1,333 (53%)	1,538 (61%)	5,143 (52%)	
Civil status **	Married/registered partner	1,258 (54%)	1,416 (55%)	1,329 (53%)	1,309 (52%)	4,654 (47%)	0.348
	Not married: never married, divorced, widowed	1,070 (46%)	1,175 (45%)	1,201 (47%)	1,208 (48%)	5,312 (53%)	
	Missing	0 (0%)	0 (0%)	1 (0%)	1 (0%)	2 (0%)	
Highest level of commenced education **	Primary school (maximum 9 years)	909 (39%)	1,011 (39%)	965 (38%)	775 (31%)	3,660 (37%)	< 0.001
	Secondary school (3–4 additional years)	945 (41%)	1,011 (39%)	993 (39%)	1,039 (41%)	3,988 (40%)	
	Higher education	459 (20%)	531 (20%)	550 (22%)	680 (27%)	2,220 (22%)	
	Missing	14 (1%)	38 (1%)	24 (1%)	24 (1%)	100 (1%)	
Disposable income per family unit divided into annual quartiles. ***	1st quartile	676 (29%)	653 (25%)	624 (25%)	543 (22%)	2,496 (25%)	< 0.001
	2nd quartile	609 (26%)	646 (25%)	648 (26%)	584 (23%)	2,487 (25%)	
	3rd quartile	569 (24%)	685 (26%)	633 (25%)	605 (24%)	2,492 (25%)	
	4th quartile	474 (20%)	607 (23%)	625 (25%)	785 (31%)	2,491 (25%)	
	Missing	0 (0%)	0 (0%)	1 (0%)	1 (0%)	2 (0%)	

Includes 9,968 patients diagnosed with metastatic colorectal cancer in 2009–2016 in Sweden

*From chi square test

**Year of/up to 3 years before if missing

***Year before diagnosis or up to 3 years before or year of if missing

The correct Table 1 is given below.

**Table 1:** Patient characteristics according to annual hospital volume of patients with metastatic colorectal cancer

		First quartile *n* = 2,402	Second quartile *n* = 2,439	Third quartile *n* = 2,433	Fourth quartile *n* = 2,694	All *n* = 9,968	*P*-value*
Sex	Male	1,352 (56%)	1,377 (56%)	1,347 (55%)	1,490 (55%)	5,566 (56%)	0.776
	Female	1,050 (44%)	1,062 (44%)	1,086 (45%)	1,204 (45%)	4,402 (44%)	
Age	Median (years)	71	70	71	69	70	
	18–64 years	646 (27%)	738 (30%)	719 (30%)	958 (36%)	3,061 (31%)	< 0.001
	65–79 years	1,221 (51%)	1,180 (48%)	1,178 (48%)	1,258 (47%)	4,837 (49%)	
	≥ 80 years	535 (22%)	521 (21%)	536 (22%)	478 (18%)	2,070 (21%)	
Charlson comorbidity index	0	1,507 (63%)	1,517(62%)	1,454 (60%)	1,657 (62%)	6,135 (62%)	0.157
	1	238 (10%)	266 (11%)	292 (12%)	274 (10%)	1,070 (11%)	
	≥ 2	657 (27%)	656 (27%)	687 (28%)	763 (28%)	2,763 (28%)	
Year of diagnosis	2009–2012	1,157 (48%)	1,173 (48%)	1,219 (50%)	1276(47%)	4,825 (48%)	0.248
	2013–2016	1,245 (52%)	1,266 (52%)	1,214 (50%)	1,418 (53%)	5,143 (52%)	
Civil status **	Married/registered partner	1,304 (54%)	1,320 (54%)	1,275 (52%)	1,413 (52%)	4,654 (47%)	0.545
	Not married: never married, divorced, widowed	1,098 (46%)	1,119 (46%)	1,157 (48%)	1,280 (48%)	5,312 (53%)	
	Missing	0 (0%)	0 (0%)	1 (0%)	1 (0%)	2 (0%)	
Highest level of commenced education **	Primary school (maximum 9 years)	921 (38%)	960 (39%)	940 (39%)	839 (31%)	3,660 (37%)	< 0.001
	Secondary school (3–4 additional years)	979 (41%)	958 (39%)	959 (39%)	1,092 (41%)	3,988 (40%)	
	Higher education	487 (20%)	490 (20%)	502 (21%)	741 (28%)	2,220 (22%)	
	Missing	15 (1%)	31 (1%)	32 (1%)	22 (1%)	100 (1%)	
Disposable income per family unit divided into annual quartiles. ***	1st quartile	677 (28%)	590 (24%)	616 (25%)	613 (22%)	2,496 (25%)	< 0.001
	2nd quartile	635 (26%)	629 (26%)	602 (25%)	621 (23%)	2,487 (25%)	
	3rd quartile	579 (24%)	654 (27%)	603 (25%)	656 (24%)	2,492 (25%)	
	4th quartile	511 (21%)	566 (23%)	611 (25%)	803 (30%)	2,491 (25%)	
	Missing	0 (0%)	0 (0%)	1 (0%)	1 (0%)	2 (0%)	

Includes 9,968 patients diagnosed with metastatic colorectal cancer in 2009–2016 in Sweden

*From chi square test

**Year of/up to 3 years before if missing

***Year before diagnosis or up to 3 years before or year of if missing

The incorrect Table 2 is given below.

**Table 2:** Hospital and tumour characteristics according to annual hospital volume of patients with metastatic colorectal cancer

		First quartile *n* = 2,328	Second quartile *n* = 2,591	Third quartile *n* = 2,531	Fourth quartile *n* = 2,518	All *n* = 9,968	*P*-value*
Hospital level	University	79 (3%)	492 (19%)	669 (26%)	1,928 (77%)	3,168 (32%)	< 0.001
	Non-university	2,249 (97%)	2,099 (81%)	1,862 (74%)	590 (23%)	6,800 (68%)	
Hospital region	1	221 (9%)	370 (14%)	635 (25%)	741 (29%)	1,967 (20%)	< 0.001
	2	505 (22%)	608 (23%)	869 (34%)	273 (11%)	2,255 (23%)	
	3	370 (16%)	449 (17%)	231 (9%)	0 (0%)	1,050 (11%)	
	4	390 (17%)	459 (18%)	407 (16%)	763 (30%)	2,019 (20%)	
	5	390 (17%)	401 (15%)	288 (11%)	741 (29%)	1,820 (18%)	
	6	452 (19%)	304 (12%)	101 (4%)	0 (0%)	857 (9%)	
Primary tumour location	Right colon	615 (26%)	637 (25%)	643 (25%)	676 (27%)	2,568 (26%)	< 0.001
	Transverse colon	314 (13%)	292 (11%)	316 (12%)	306 (12%)	1,227 (12%)	
	Left colon	743 (32%)	761 (29%)	732 (29%)	673 (27%)	2,912 (29%)	
	Unknown location in colon	21 (1%)	19 (1%)	9 (0%)	19 (1%)	68 (1%)	
	Rectum	625 (27%)	882 (34%)	831 (33%)	844 (34%)	3,193 (32%)	
Pretherapeutic multidisciplinary team conference	Yes	1,507 (65%)	1,967 (76%)	1,950 (77%)	2,011 (80%)	7,435 (75%)	< 0.001
	No	812 (35%)	621 (24%)	570 (23%)	498 (20%)	2,510 (25%)	
	Missing	8 (0%)	3 (0%)	3 (0%)	9 (0%)	23 (0%)	
Resection of primary tumour	No	1,128 (48%)	1,349 (52%)	1,397 (55%)	1,322 (53%)	5,196 (52%)	< 0.001
	Yes	1,199 (52%)	1,242 (48%)	1,135 (45%)	1,196 (47%)	4,772 (48%)	
cT	1–3	659 (28%)	959 (37%)	824 (33%)	824 (34%)	3,264 (33%)	< 0.001
	4	564 (24%)	757 (29%)	735 (29%)	795 (32%)	2,849 (29%)	
	X	619 (27%)	485 (19%)	500 (20%)	611 (24%)	2,218 (22%)	
	Missing	486 (21%)	290 (15%)	472 (19%)	288 (11%)	1,637 (16%)	
cN	0	450 (19%)	531 (20%)	413 (16%)	401 (16%)	1,795 (18%)	< 0.001
	1–2	1,099 (47%)	1,434 (55%)	1,521 (60%)	1,362 (54%)	5,414 (54%)	
	X	660 (28%)	543 (21%)	540 (21%)	702 (28%)	2,447 (25%)	
	Missing	119 (5%)	83 (3%)	57 (2%)	53 (2%)	312 (3%)	
Metastatic location	Liver	744 (32%)	913 (35%)	908 (36%)	805 (32%)	3,370 (34%)	< 0.001
	Lung	205 (9%)	176 (7%)	170 (7%)	128 (5%)	679 (7%)	
	Peritoneum	62 (3%)	70 (3%)	85 (3%)	111 (4%)	328 (3%)	
	Other	73 (3%)	93 (4%)	83 (3%)	105 (4%)	354 (4%)	
	Multiple locations	1,244 (53%)	1,339 (52%)	1,285 (51%)	1,369 (54%)	5,237 (53%)	

Includes 9,968 patients diagnosed with metastatic colorectal cancer in 2009–2016 in Sweden

*From chi square test

*Abbreviations:* *cN* clinical nodal status, *CT* clinical tumour status

The correct Table 2 is given below

**Table 2:** Hospital and tumour characteristics according to annual hospital volume of patients with metastatic colorectal cancer

		First quartile *n* = 2,402	Second quartile *n* = 2,439	Third quartile *n* = 2,433	Fourth quartile *n* = 2,694	All *n* = 9,968	*P*-value*
Hospital level	University	105 (4%)	356 (15%)	537 22%)	2,170 81%)	3,168 (32%)	< 0.001
	Non-university	2,297 (96%)	2,083 (85%)	1,896 (78%)	524 (19%)	6,800 (68%)	
Hospital region	1	221 (9%)	281 (12%)	592 (24%)	873 (32%)	1,967 (20%)	< 0.001
	2	453 (19%)	706 (29%)	762 (31%)	334 (12%)	2,255 (23%)	
	3	373 (16%)	489 (20%)	101 (4%)	87(3%)	1,050 (11%)	
	4	395 (16%)	368 (15%)	543 (22%)	713 (26%)	2,019 (20%)	
	5	439 (18%)	357 (15%)	337 (14%)	687 (26%)	1,820 (18%)	
	6	439 (18%)	238 (10%)	98 (4%)	0 (0%)	857 (9%)	
Primary tumour location	Right colon	647 (27%)	577 (24%)	630 (26%)	717 (27%)	2,568 (26%)	< 0.001
	Transverse colon	314 (13%)	274 (11%)	317 (13%)	323 (12%)	1,227 (12%)	
	Left colon	770 (32%)	711 (29%)	705 (29%)	723 (27%)	2,912 (29%)	
	Unknown location in colon	21 (1%)	19 (1%)	9 (0%)	19 (1%)	68 (1%)	
	Rectum	650 (27%)	858 (35%)	772 (32%)	912 (34%)	3,193 (32%)	
Pretherapeutic multidisciplinary team conference	Yes	1,607 (67%)	1,852 (76%)	1,859 (76%)	2,117 (79%)	7,435 (75%)	< 0.001
	No	787 (33%)	584 (24%)	572 (24%)	567 (21%)	2,510 (25%)	
	Missing	8 (0%)	3 (0%)	2 (0%)	10(0%)	23 (0%)	
Resection of primary tumour	No	1,174 (48%)	1,297 (53%)	1,360 (56%)	1,365 (51%)	5,196 (52%)	< 0.001
	Yes	1,228 (51%)	1,142 (47%)	1,073 (44%)	1,329 (49%)	4,772 (48%)	
cT	1–3	696 (29%)	912 (37%)	812 (33%)	846 (34%)	3,266 (33%)	< 0.001
	4	598 (25%)	713 (29%)	721 (30%)	819 (31%)	2,851 (29%)	
	X	637 (27%)	445 (18%)	481 (20%)	652 (24%)	2,218 (22%)	
	Missing	471 (20%)	369 (15%)	419 (17%)	377 (14%)	1,637 (16%)	
cN	0	475 (20%)	469 (19%)	447 (18%)	404 (15%)	1,795 (18%)	< 0.001
	1–2	1,170 (49%)	1,372 (56%)	1,433 (59%)	1,441 (53%)	5,414 (54%)	
	X	641 (27%)	520 (21%)	502 (21%)	782 (29%)	2,447 (25%)	
	Missing	116 (5%)	78 (3%)	51 (2%)	67 (2%)	312 (3%)	
Metastatic location	Liver	772 (32%)	826 (34%)	879 (36%)	893 (33%)	3,370 (34%)	< 0.001
	Lung	210 (9%)	177 (7%)	159 (7%)	133 (5%)	679 (7%)	
	Peritoneum	68 (3%)	55 (2%)	86 (4%)	119 (4%)	328 (3%)	
	Other	77 (3%)	91 (4%)	72 (3%)	114 (4%)	354 (4%)	
	Multiple locations	1,275 (53%)	1,290 (53%)	1,237 (51%)	1,435 (53%)	5,237 (53%)	

Includes 9,968 patients diagnosed with metastatic colorectal cancer in 2009–2016 in Sweden

*From chi square test

*Abbreviations*: *cN* clinical nodal status, *CT* clinical tumour status

The incorrect Table 3 is given below

**Table 3:** Cox regression model estimates of all-cause mortality hazard ratios (HRs) with 95% confidence intervals (CIs)

		**Univariable**	**First model**.^**1**^	**Second model**.^**2**^
		HR* (95% CI)	HR* (95% CI)	HR* (95% CI)
Hospital mCRC case load	1^st^ quartile	1	1	1
	2^nd^ quartile	**0.93 (0.87–0.99)**	1.01 (0.95–1.07)	1.04 (0.98–1.11)
	3^rd^ quartile	0.97 (0.92–1.04)	1.02 (0.96–1.09)	**1.07 (1.00–1.14)**
	4^th^ quartile	**0.87 (0.82–0.93)**	**0.92 (0.86–0.98)**	1.06 (0.98–1.14)
Hospital level	Non-university	1	1	1
	University	**0.81 (0.78–0.85)**	**0.85 (0.81–0.89)**	**0.83 (0.78–0.88)**

Model includes 9,968 patients with synchronous colorectal cancer metastases

*Adjusted for time since diagnosis

^1^One exposure (hospital volume/ hospital level) and adjusted for sex (male, female), age (categorized: 18–64,65–79, ≥ 80 years), Charlson comorbidity index (0,1, ≥ 2), Primary tumour location (right colon, transverse colon, left colon, colon unknown location in colon, rectum), cT (1–3, 4, X) cN, (0, 1–2, X), metastases (single, multiple), civil status (married, not married), education (primary, secondary, higher education), annual income (quartiles), year of diagnosis. Missing data handled using missing indicator method

^2^Model of both exposures, adjusted as above

The correct Table 3 is given below.

**Table 3:** Cox regression model estimates of all-cause mortality hazard ratios (HRs) with 95% confidence intervals (CIs)

		**Univariable**	**First model **^**1**^	**Second model**^**2**^
		HR* (95% CI)	HR* (95% CI)	HR* (95% CI)
Hospital mCRC case load	1^st^ quartile	1	1	1
	2^nd^ quartile	**0.94 (0.88–1.00)**	1.00 (0.94–1.07)	1.02 (0.96–1.09)
	3^rd^ quartile	1.00 (0.95–1.07)	1.06 (0.99–1.12)	**1.09 (1.02–1.16)**
	4^th^ quartile	**0.87 (0.82–0.93)**	**0.90 (0.85–0.96)**	1.04 (0.96–1.12)
Hospital level	Non-university	1	1	1
	University	**0.81 (0.78–0.85)**	**0.85 (0.81–0.89)**	**0.84 (0.79–0.89)**

Model includes 9,968 patients with synchronous colorectal cancer metastases

*Adjusted for time since diagnosis

^1^One exposure (hospital volume/ hospital level) and adjusted for sex (male, female), age (categorized: 18–64,65–79, ≥ 80 years), Charlson comorbidity index (0,1, ≥ 2), Primary tumour location (right colon, transverse colon, left colon, colon unknown location in colon, rectum), cT (1–3, 4, X) cN, (0, 1–2, X), metastases (single, multiple), civil status (married, not married), education (primary, secondary, higher education), annual income (quartiles), year of diagnosis. Missing data handled using missing indicator method

^2^Model of both exposures, adjusted as above

The incorrect Supplementary Table 2 is given below.

Supplementary Table 2. Additional treatment-associated patient characteristics for 9,968 patients with synchronous metastatic colorectal cancer (mCRC) according to the managing hospitals’ annual incident number of patients with mCRC divided into 4 quartiles.

**Table:** 

		1st quartile n = 2,328	2nd quartile n = 2,591	3rd quartile n = 2,531	4th quartile n = 2,518	All n = 9,968	P-value
ASA class	1	159 (7%)	148 (6%)	165 (7%)	117 (5%)	589 (6%)	< 0.001
	2	622 (27%)	668 (26%)	607 (24%)	619 (25%)	2,516 (25%)	
	3	386 (17%)	386 (15%)	395 (16%)	493 (20%)	1,660 (17%)	
	4–5	59 (3%)	30 (1%)	51 (2%)	61 (2%)	201 (2%)	
	Missing	1,102 (47%)	1,359 (52%)	1,313 (52%)	1,228 (49%)	5,002 (50%)	
Neoadjuvant treatment for metastatic colon cancer*	No	1,476 (87%)	1 431 (84%)	1 457 (86%)	1 303 (78%)	5,667 (84%)	< 0.001
	Yes	200 (12%)	266 (16%)	233 (14%)	254 (15%)	952 (14%)	
	Missing	17 (1%)	12 (1%)	10 (1%)	117 (7%)	156 (2%)	
Neoadjuvant treatment for metastatic rectal cancer*	No	387 (61%)	458 (52%)	453 (55%)	411 (49%)	411 (49%)	< 0.001
	Chemotherapy	77 (12%)	76 (9%)	69 (8%)	45 (5%)	267 (8%)	
	Radiotherapy	63 (10%)	119 (14%)	107 (13%)	96 (11%)	385 (12%)	
	Combination	95 (15%)	220 (25%)	194 (23%)	240 (28%)	750 (23%)	
	Missing	13 (2%)	9 (1%)	8 (1%)	52 (6%)	82 (3%)	
pT (if resected)	1–3	630 (53%)	672 (54%)	606 (53%)	502 (42%)	2,410 (51%)	< 0.001
	4	452 (38%)	425 (34%)	397 (35%)	539 (45%)	1,813 (38%)	
	X/Missing	117 (10%)	145 (12%)	132 (12%)	155 (13%)	549 (12%)	
pN (if resected)	0	234 (20%)	249 (20%)	268 (24%)	242 (20%)	993 (21%)	0.003
	1	334 (28%)	382 (31%)	332 (29%)	383 (32%)	1,431 (30%)	
	2	507 (42%)	462 (37%)	404 (36%)	414 (35%)	1,787 (37%)	
	X/missing	124 (10%)	149 (12%)	131 (12%)	157 (13%)	561 (12%)	
Surgery of metastases (with or without primary tumour resection)	Liver**	263 (11%)	412 (16%)	375 (15%)	366 (15%)	1,416 (14%)	< 0.001
	Lung**	23 (1%)	29 (1%)	34 (1%)	42 (2%)	128 (1%)	
	Peritoneal	30 (1%)	17 (1%)	28 (1%)	125 (5%)	200 (2%)	
	Combination**	9 (0%)	10 (0%)	9 (0%)	25 (1%)	53 (1%)	
	No	2,002 (86%)	2,123 (82%)	2,086 (82%)	1,960 (78%)	8,171 (82%)	

*Data unspecified on whether the neoadjuvant treatment was given for the primary tumour or the metastases or both

**A total of 46 patients who underwent surgery for liver metastases (of which one had combination metastatic surgery) received solely non-surgical locally ablative therapy (percutaneous destruction or embolisation treatment) and four patients with lung metastases had solely non-surgical treatment (stereotactic body radiation, SBRT). The solely non-surgical locally ablative treatments constituted 3% (n = 50/1797) of the patients treated with metastatic surgery

Abbreviations: ASA (American Society of Anaesthesiologists), pN (pathological nodal status), pT (pathological tumour status).

The correct Supplementary Table 2 is given below.

Supplementary Table 2. Additional treatment-associated patient characteristics for 9,968 patients with synchronous metastatic colorectal cancer (mCRC) according to the managing hospitals’ annual incident number of patients with mCRC divided into 4 quartiles.

**Table:** 

		1st quartile n = 2,402	2nd quartile n = 2,439	3rd quartile n = 2,433	4th quartile n = 2,694	All n = 9,968	P-value
ASA class	1	153 (6%)	135 (6%)	156 (6%)	145 (5%)	589 (6%)	< 0.001
	2	627 (26%)	624 (26%)	584 (24%)	681 (25%)	2,516 (25%)	
	3	398 (17%)	349 (14%)	375 (15%)	538 (20%)	1,660 (17%)	
	4–5	54 (2%)	33 (1%)	45 (2%)	69 (3%)	201 (2%)	
	Missing	1,179 (49%)	1,359 (52%)	1,273 (52%)	1,228 (49%)	5,002 (50%)	
Neoadjuvant treatment for metastatic colon cancer*	No	1,476 (87%)	1 431 (84%)	1 457 (86%)	1 303 (78%)	5,667 (84%)	< 0.001
	Yes	200 (12%)	266 (16%)	233 (14%)	254 (15%)	952 (14%)	
	Missing	17 (1%)	12 (1%)	10 (1%)	117 (7%)	156 (2%)	
Neoadjuvant treatment for metastatic rectal cancer*	No	400 (62%)	445 (52%)	448 (58%)	416 (46%)	1709 (54%)	< 0.001
	Chemotherapy	76 (12%)	67 (8%)	62 (8%)	62 (7%)	267 (8%)	
	Radiotherapy	61 (9%)	115 (13%)	93 (12%)	116 (13%)	385 (12%)	
	Combination	103 (16%)	220 (26%)	160 (21%)	266 (29%)	749 (23%)	
	Missing	10 (2%)	11 (1%)	9 (1%)	52 (6%)	82 (3%)	
pT (if resected)	1–3	641 (52%)	638 (56%)	544 (51%)	588 (44%)	2,411 (51%)	< 0.001
	4	458 (37%)	365 (32%)	413 (38%)	576 (43%)	1,812 (38%)	
	X/Missing	129 (11%)	139 (12%)	116 (11%)	165 (12%)	549 (12%)	
pN (if resected)	0	234 (19%)	240 (21%)	240 (22%)	282 (21%)	996 (21%)	0.141
	1	358 (29%)	341 (30%)	309 (29%)	421 (32%)	1,429 (30%)	
	2	501 (41%)	420 (37%)	404 (38%)	461 (35%)	1,786 (37%)	
	X/missing	135 (11%)	141 (12%)	120 (11%)	165 (12%)	561 (12%)	
Surgery of metastases (with or without primary tumour resection)	Liver**	273 (11%)	377 (15%)	348 (14%)	418 (16%)	1,416 (14%)	< 0.001
	Lung**	24 (1%)	28 (1%)	39 (2%)	37 (1%)	128 (1%)	
	Peritoneal	31 (1%)	17 (1%)	25 (1%)	127 (5%)	200 (2%)	
	Combination**	10 (0%)	7 (0%)	10 (0%)	26 (1%)	53 (1%)	
	No	2,064 (86%)	2,010 (82%)	2,011 (83%)	2,086 (77%)	8,171 (82%)	

*Data unspecified on whether the neoadjuvant treatment was given for the primary tumour or the metastases or both

**A total of 46 patients who underwent surgery for liver metastases (of which one had combination metastatic surgery) received solely non-surgical locally ablative therapy (percutaneous destruction or embolisation treatment) and four patients with lung metastases had solely non-surgical treatment (stereotactic body radiation, SBRT). The solely non-surgical locally ablative treatments constituted 3% (n = 50/1797) of the patients treated with metastatic surgery

Abbreviations: ASA (American Society of Anaesthesiologists), pN (pathological nodal status), pT (pathological tumour status)

The incorrect Supplementary Table 3 is given below.

Supplementary Table 3. Summary table of results.

**Table:** 

Analysis	Outcome	Exposure		Results
Multivariable logistic regression adjusted	Metastatic surgery	Hospital volume in quartiles (qts)	2nd qt vs 1st qt	OR (95% CI): **1.28 (1.08–1.52)**
			3rd qt vs 1st qt	OR (95% CI):** 1.33 (1.12–1.58)**
			4th qt vs 1st qt	OR (95% CI):** 1.68 (1.42–1.98)**
Multivariable logistic regression adjusted	Metastatic surgery	Hospital level	University vs non-university	OR (95% CI): **1.89 (1.68–2.12)**
Multivariable logistic regression adjusted for both exposures	Metastatic surgery	Hospital volume in quartiles (qts) Hospital level	2nd qt vs 1st qt	OR (95% CI): 1.14 (0.96–1.36)
			3rd qt vs 1st qt	OR (95% CI): 1.13(0.94–1.34)
			4th qt vs 1st qt	OR (95% CI): 1 (0.82–1.23)
			University vs non-university	OR (95% CI):** 1.94 (1.68–2.24)**
	Interaction	Hospital volume and hospital level		Wald test p < 0.001
Multivariable logistic regression adjusted for both exposures and mediator MDT	Metastatic surgery	Hospital volume in quartiles (qts) Hospital level mediator MDT	2nd qt vs 1st qt	OR (95% CI): 1.13 (0.95–1.34)
			3rd qt vs 1st qt	OR (95% CI): 1.10 (0.92–1.32)
			4th qt vs 1st qt	OR (95% CI): 1 (0.81–1.22)
			University vs non-university	OR (95% CI):** 1.90 (1.64–2.19)**
			Yes vs no	OR (95% CI):** 1.54 (1.30–1.82)**
	Interaction	MDT and hospital level		Wald test p < 0.001
Multivariable Cox regression model adjusted	All-cause mortality	Hospital volume in qts	2nd qt vs 1st qt	HR (95% CI): 1.01 (0.95–1.07)
			3rd qt vs 1st qt	HR (95% CI): 1.02 (0.96–1.09)
			4th qt vs 1st qt	HR (95% CI): **0.92 (0.86–0.98)**
Multivariable Cox regression model adjusted	All-cause mortality	Hospital level	University vs non-university	HR (95% CI): **0.85 (0.81–0.89)**
Multivariable Cox regression model adjusted for both exposures	All-cause mortality	Hospital volume in quartiles (qts) Hospital level	2nd qt vs 1st qt	HR (95% CI): 1.04 (0.98–1.11)
			3rd qt vs 1st qt	HR (95% CI): **1.07 (1.00–1.14)**
			4th qt vs 1st qt	HR (95% CI): 1.06 (0.98–1.14)
			University vs non-university	HR (95% CI): **0.83 (0.78- 0.88)**

All multivariable models were adjusted for sex (male, female), age (18–64,65–79, ≥ 80 years), Charlson comorbidity index (0,1, ≥ 2), Primary tumour location (right colon, transverse colon, left colon, colon unknown location in colon, rectum), cT-stage (1–3, 4, X), cN-stage (0, 1–2, X), metastases (single, multiple), civil status (married, not married), education (primary, secondary, higher education), income (4 annual quartiles), diagnosis year (continuous 2009–2016). Missing data handled using missing indicator method. Multivariable logistic regression models included 9,966 patients. Multivariable cox regression models included 9,968 patients

Abbreviations: CI: confidence interval, HR: hazard ratio, MDT: multidisciplinary team conference, OR: odds ratio, qt: quartile

The correct Supplementary Table 3 is given below.

Supplementary Table 3. Summary table of results.

**Table:** 

Analysis	Outcome	Exposure		Results
Multivariable logistic regression adjusted	Metastatic surgery	Hospital volume in quartiles (qts)	2nd qt vs 1st qt	OR (95% CI): **1.23 (1.03–1.46)**
			3rd qt vs 1st qt	OR (95% CI): **1.28 (1.08–1.52)**
			4th qt vs 1st qt	OR (95% CI): **1.78 (1.52–2.09)**
Multivariable logistic regression adjusted	Metastatic surgery	Hospital level	University vs non-university	OR (95% CI): **1.89 (1.68–2.12)**
Multivariable logistic regression adjusted for both exposures	Metastatic surgery	Hospital volume in quartiles (qts) Hospital level	2nd qt vs 1st qt	OR (95% CI): 1.15 (0.96–1.36)
			3rd qt vs 1st qt	OR (95% CI): 1.14(0.95–1.35)
			4th qt vs 1st qt	OR (95% CI): 1.10 (0.90–1.35)
			University vs non-university	OR (95% CI): **1.86 (1.59–2.16)**
	Interaction	Hospital volume and hospital level		Wald test p < 0.001
Multivariable logistic regression adjusted for both exposures and mediator MDT	Metastatic surgery	Hospital volume in quartiles (qts) Hospital level mediator MDT	2nd qt vs 1st qt	OR (95% CI): 1.13 (0.95–1.34)
			3rd qt vs 1st qt	OR (95% CI): 1.12 (0.94–1.33)
			4th qt vs 1st qt	OR (95% CI): 1.09 (0.89–1.33)
			University vs non-university	OR (95% CI): **1.82 (1.56–2.12)**
			Yes vs no	OR (95% CI):** 1.54 (1.30–1.82)**
	Interaction	MDT and hospital level		Wald test p < 0.001
Multivariable Cox regression model adjusted	All-cause mortality	Hospital volume in qts	2nd qt vs 1st qt	HR (95% CI): 1.00 (0.94–1.07)
			3rd qt vs 1st qt	HR (95% CI): 1.06 (0.99–1.12)
			4th qt vs 1st qt	HR (95% CI): **0.90 (0.85–0.96)**
Multivariable Cox regression model adjusted	All-cause mortality	Hospital level	University vs non-university	HR (95% CI): **0.85 (0.81–0.89)**
Multivariable Cox regression model adjusted for both exposures	All-cause mortality	Hospital volume in quartiles (qts) Hospital level	2nd qt vs 1st qt	HR (95% CI): 1.02 (0.96–1.09)
			3rd qt vs 1st qt	HR (95% CI): **1.09 (1.02–1.16)**
			4th qt vs 1st qt	HR (95% CI): 1.04 (0.96–1.12)
			University vs non-university	HR (95% CI): **0.84 (0.79- 0.89)**

All multivariable models were adjusted for sex (male, female), age (18–64,65–79, ≥ 80 years), Charlson comorbidity index (0,1, ≥ 2), Primary tumour location (right colon, transverse colon, left colon, colon unknown location in colon, rectum), cT-stage (1–3, 4, X), cN-stage (0, 1–2, X), metastases (single, multiple), civil status (married, not married), education (primary, secondary, higher education), income (4 annual quartiles), diagnosis year (continuous 2009–2016). Missing data handled using missing indicator method. Multivariable logistic regression models included 9,966 patients. Multivariable cox regression models included 9,968 patients

Abbreviations: CI: confidence interval, HR: hazard ratio, MDT: multidisciplinary team conference, OR: odds ratio, qt: quartile
