# Supplementary material for: Hospital factors and metastatic surgery in colorectal cancer patients, a population-based cohort study
Source: BMC Cancer. 2022 Aug 19;22:907. doi: 10.1186/s12885-022-10005-8 (PMC9392345; doi:10.1186/s12885-022-10005-8)
Supplement: Supplementary file 1 — Additional file 1: Supplementary table 1. Treatment codes as described by the National Board of Health and Welfare in Sweden used to identify potentially curative treatments of metastases and primary tumour, translated from Swedish.*Defined as non-surgical treatment. Supplementary table 2. Additional treatment-associated patient characteristics for 9,968 patients with synchronous metastatic colorectal cancer (mCRC) according to the managing hospitals’ annual incident number of patients with mCRC divided into 4 quartiles. *Data unspecified on whether the neoadjuvant treatment was given for the primary tumour or the metastases or both. ** A total of 46 patients who underwent surgery for liver metastases (of which one had combination metastatic surgery) received solely non-surgical locally ablative therapy (percutaneous destruction or embolisation treatment) and four patients with lung metastases had solely non-surgical treatment (stereotactic body radiation, SBRT). The solely non-surgical locally ablative treatments constituted 3% (n=50/1797) of the patients treated with metastatic surgery. Abbreviations: ASA (American Society of Anaesthesiologists), pN (pathological nodal status), pT (pathological tumour status). Supplementary table 3. Summary table of results. All multivariable models were adjusted for sex (male, female), age (18-64,65-79, ≥80 years), Charlson comorbidity index (0,1,≥2), Primary tumour location (right colon, transverse colon, left colon, colon unknown location in colon, rectum), cT-stage (1-3, 4, X) , cN-stage (0, 1-2, X), metastases (single, multiple), civil status (married, not married), education (primary, secondary, higher education), income (4 annual quartiles), diagnosis year (continuous 2009-2016). Missing data handled using missing indicator method. Multivariable logistic regression models included 9,966 patients. Multivariable cox regression models included 9,968 patients. Abbreviations: CI: confidence interval, HR: hazard ratio, MDT: multi [file 12885_2022_10005_MOESM1_ESM.docx]

Supplementary table 1. Treatment codes as described by the National Board of Health and Welfare in Sweden used to identify potentially curative treatments of metastases and primary tumour, translated from Swedish

| **Primary tumour** | |
| --- | --- |
| JEA | Appendectomy |
| JFA83-85 | Extirpation of local growth in colon, endoscopic mucosal or submucosal resection of colon |
| JFB2-6 | Resection of colon |
| JFB9 | Other resection of small bowel or colon, open or laparoscopic |
| JFH | Colectomy |
| JGA | Proctectomy or local rectal procedure |
| JGB | Resection and extirpation of rectum |
| JGW | Other surgery of rectum |
| **Liver** | |
| JJA40 | Excision of growth in the liver |
| JJA41 | Laparoscopic excision of growth in the liver |
| JJA43 | Destruction of growth in the liver |
| JJA44 | Laparoscopic destruction of growth in the liver |
| JJA96 | Other local procedure of the liver |
| JJA97 | Other laparoscopic local procedure of the liver |
| JJB- | Liver resections |
| JJW96 | Other procedure of liver |
| TJJ10* | Percutaneous destruction of growth in liver |
| DJ026* | Percutaneous transluminal injection of Yttrium-90 microspheres in the liver |
| **Lungs** | |
| GDA20 | Extirpation of local growth in the lungs |
| GDA21 | Thoracoscopic extirpation of local growth in the lungs |
| GDB- | Lung resections |
| GDC- | Lung lobectomies |
| GDD- | Pulmectomies |
| ZV520* | Stereotactic radiation (non-intracranial) |
| **Peritoneum** | |
| JAL20 | Extirpation or destruction of peritoneal growth |
| JAW96 | Other surgery on abdominal wall, mesentery, peritoneum or omentum. |
| JAQ00 | Extensive extirpation of peritoneum, total or subtotal extirpation at certain metastatic conditions. |
| JAQ10 | Intraoperative hyperthermic chemotherapeutic washing of the abdominal cavity. Adjuvant therapy after extensive extirpation of the peritoneum. |

*Defined as non-surgical treatment

Supplementary table 2. Additional treatment-associated patient characteristics for 9,968 patients with synchronous metastatic colorectal cancer (mCRC) according to the managing hospitals’ annual incident number of patients with mCRC divided into 4 quartiles

|  |  | 1st quartile  n=2,328 | 2nd quartile  n=2,591 | 3rd quartile  n=2,531 | 4th quartile  n=2,518 | All n=9,968 | P-value |
| --- | --- | --- | --- | --- | --- | --- | --- |
| ASA class | 1 | 159 (7%) | 148 (6%) | 165 (7%) | 117 (5%) | 589 (6%) | <0.001 |
|  | 2 | 622 (27%) | 668 (26%) | 607 (24%) | 619 (25%) | 2,516 (25%) |  |
|  | 3 | 386 (17%) | 386 (15%) | 395 (16%) | 493 (20%) | 1,660 (17%) |  |
|  | 4-5 | 59 (3%) | 30 (1%) | 51 (2%) | 61 (2%) | 201 (2%) |  |
|  | Missing | 1,102 (47%) | 1,359 (52%) | 1,313 (52%) | 1,228 (49%) | 5,002 (50%) |  |
| Neoadjuvant treatment for metastatic colon cancer* | No | 1,476 (87%) | 1 431 (84%) | 1 457 (86%) | 1 303 (78%) | 5,667 (84%) | <0.001 |
|  | Yes | 200 (12%) | 266 (16%) | 233 (14%) | 254 (15%) | 952 (14%) |  |
|  | Missing | 17 (1%) | 12 (1%) | 10 (1%) | 117 (7%) | 156 (2%) |  |
| Neoadjuvant treatment for metastatic rectal cancer* | No | 387 (61%) | 458 (52%) | 453 (55%) | 411 (49%) | 411 (49%) | <0.001 |
|  | Chemotherapy | 77 (12%) | 76 (9%) | 69 (8%) | 45 (5%) | 267 (8%) |  |
|  | Radiotherapy | 63 (10%) | 119 (14%) | 107 (13%) | 96 (11%) | 385 (12%) |  |
|  | Combination | 95 (15%) | 220 (25%) | 194 (23%) | 240 (28%) | 750 (23%) |  |
|  | Missing | 13 (2%) | 9 (1%) | 8 (1%) | 52 (6%) | 82 (3%) |  |
| pT (if resected) | 1-3 | 630 (53%) | 672 (54%) | 606 (53%) | 502 (42%) | 2,410 (51%) | <0.001 |
|  | 4 | 452 (38%) | 425 (34%) | 397 (35%) | 539 (45%) | 1,813 (38%) |  |
|  | X/Missing | 117 (10%) | 145 (12%) | 132 (12%) | 155 (13%) | 549 (12%) |  |
| pN (if resected) | 0 | 234 (20%) | 249 (20%) | 268 (24%) | 242 (20%) | 993 (21%) | 0.003 |
|  | 1 | 334 (28%) | 382 (31%) | 332 (29%) | 383 (32%) | 1,431 (30%) |  |
|  | 2 | 507 (42%) | 462 (37%) | 404 (36%) | 414 (35%) | 1,787 (37%) |  |
|  | X/missing | 124 (10%) | 149 (12%) | 131 (12%) | 157 (13%) | 561 (12%) |  |
| Surgery of metastases (with or without primary tumour resection) | Liver** | 263 (11%) | 412 (16%) | 375 (15%) | 366 (15%) | 1,416 (14%) | <0.001 |
|  | Lung** | 23 (1%) | 29 (1%) | 34 (1%) | 42 (2%) | 128 (1%) |  |
|  | Peritoneal | 30 (1%) | 17 (1%) | 28 (1%) | 125 (5%) | 200 (2%) |  |
|  | Combination** | 9 (0%) | 10 (0%) | 9 (0%) | 25 (1%) | 53 (1%) |  |
|  | No | 2,002 (86%) | 2,123 (82%) | 2,086 (82%) | 1,960 (78%) | 8,171 (82%) |  |

*Data unspecified on whether the neoadjuvant treatment was given for the primary tumour or the metastases or both.

** A total of 46 patients who underwent surgery for liver metastases (of which one had combination metastatic surgery) received solely non-surgical locally ablative therapy (percutaneous destruction or embolisation treatment) and four patients with lung metastases had solely non-surgical treatment (stereotactic body radiation, SBRT). The solely non-surgical locally ablative treatments constituted 3% (n=50/1797) of the patients treated with metastatic surgery.

Abbreviations: ASA (American Society of Anaesthesiologists), pN (pathological nodal status), pT (pathological tumour status).

Supplementary table 3. Summary table of results

| Analysis | Outcome | Exposure | | Results |
| --- | --- | --- | --- | --- |
| Multivariable logistic regression adjusted | Metastatic surgery | Hospital volume in quartiles (qts) | 2^nd^ qt vs 1^st^ qt | OR (95% CI): **1.28 (1.08-1.52)** |
|  |  |  | 3^rd^ qt vs 1^st^ qt | OR (95% CI): **1.33 (1.12-1.58)** |
|  |  |  | 4^th^ qt vs 1^st^ qt | OR (95% CI): **1.68 (1.42-1.98)** |
| Multivariable logistic regression adjusted | Metastatic surgery | Hospital level | University vs non-university | OR (95% CI): **1.89 (1.68-2.12)** |
| Multivariable logistic regression adjusted for both exposures | Metastatic surgery | Hospital volume in quartiles (qts) Hospital level | 2^nd^ qt vs 1^st^ qt | OR (95% CI): 1.14 (0.96-1.36) |
|  |  |  | 3^rd^ qt vs 1^st^ qt | OR (95% CI): 1.13(0.94-1.34) |
|  |  |  | 4^th^ qt vs 1^st^ qt | OR (95% CI): 1 (0.82-1.23) |
|  |  |  | University vs non-university | OR (95% CI): **1.94 (1.68-2.24)** |
|  | Interaction | Hospital volume and hospital level | | Wald test p<0.001 |
| Multivariable logistic regression adjusted for both exposures and mediator MDT | Metastatic surgery | Hospital volume in quartiles (qts) Hospital level mediator MDT | 2^nd^ qt vs 1^st^ qt | OR (95% CI): 1.13 (0.95-1.34) |
|  |  |  | 3^rd^ qt vs 1^st^ qt | OR (95% CI): 1.10 (0.92-1.32) |
|  |  |  | 4^th^ qt vs 1^st^ qt | OR (95% CI): 1 (0.81-1.22) |
|  |  |  | University vs non-university | OR (95% CI): **1.90 (1.64-2.19)** |
|  |  |  | Yes vs no | OR (95% CI): **1.54 (1.30-1.82)** |
|  | Interaction | MDT and hospital level | | Wald test p<0.001 |
| Multivariable Cox regression model adjusted | All-cause mortality | Hospital volume in qts | 2^nd^ qt vs 1^st^ qt | HR (95% CI): 1.01 (0.95-1.07) |
|  |  |  | 3^rd^ qt vs 1^st^ qt | HR (95% CI): 1.02 (0.96-1.09) |
|  |  |  | 4^th^ qt vs 1^st^ qt | HR (95% CI): **0.92 (0.86-0.98)** |
| Multivariable Cox regression model adjusted | All-cause mortality | Hospital level | University vs non-university | HR (95% CI): **0.85 (0.81-0.89)** |
| Multivariable Cox regression model adjusted for both exposures | All-cause mortality | Hospital volume in quartiles (qts) Hospital level | 2^nd^ qt vs 1^st^ qt | HR (95% CI): 1.04 (0.98-1.11) |
|  |  |  | 3^rd^ qt vs 1^st^ qt | HR (95% CI): **1.07 (1.00-1.14)** |
|  |  |  | 4^th^ qt vs 1^st^ qt | HR (95% CI): 1.06 (0.98-1.14) |
|  |  |  | University vs non-university | HR (95% CI): **0.83 (0.78- 0.88)** |

All multivariable models were adjusted for sex (male, female), age (18-64,65-79, ≥80 years), Charlson comorbidity index (0,1,≥2), Primary tumour location (right colon, transverse colon, left colon, colon unknown location in colon, rectum), cT-stage (1-3, 4, X) , cN-stage (0, 1-2, X), metastases (single, multiple), civil status (married, not married), education (primary, secondary, higher education), income (4 annual quartiles), diagnosis year (continuous 2009-2016). Missing data handled using missing indicator method. Multivariable logistic regression models included 9,966 patients. Multivariable cox regression models included 9,968 patients.

Abbreviations: CI: confidence interval, HR: hazard ratio, MDT: multidisciplinary team conference, OR: odds ratio, qt: quartile
